# Thermophysical Behavior and Molecular Interactions of Caffeine in a Novel Menthol–Resorcinol Type V Deep Eutectic Solvent

**DOI:** 10.3390/molecules31173039

**Published:** 2026-08-29

**Authors:** Milan Vraneš, Snežana Papović, Janez Cerar, Romana Cerc Korošec, Teona Teodora Borović, Jasmin Suljagić, Edita Bjelić, Mersiha Suljkanović, David Nedeljković

**Affiliations:** 1 Department of Chemistry, Biochemistry and Environmental Protection, Faculty of Sciences, University of Novi Sad, Trg Dositeja Obradovića 3, 21000 Novi Sad, Serbia; snezana.papovic@dh.uns.ac.rs (S.P.); teona@dh.uns.ac.rs (T.T.B.); david.nedeljkovic@dh.uns.ac.rs (D.N.); 2 Faculty of Chemistry and Chemical Technology, University of Ljubljana, Večna pot 113, 1000 Ljubljana, Slovenia; janez.cerar@fkkt.uni-lj.si (J.C.); romana.cerc-korosec@fkkt.uni-lj.si (R.C.K.); 3 Faculty of Technology, University of Tuzla, Urfeta Vejzagića 8, 75000 Tuzla, Bosnia and Herzegovina; jasmin.suljagic@untz.ba (J.S.); edita.bjelic@untz.ba (E.B.); 4 Faculty of Natural Sciences and Mathematics, University of Tuzla, Urfeta Vejzagića 4, 75000 Tuzla, Bosnia and Herzegovina; mersiha.suljkanovic@untz.ba

**Keywords:** density, viscosity, speed of sound, volumetric properties, TG/DSC measurements, DFT calculations

## Abstract

A nonionic type V deep eutectic solvent (DES) combining hydrophobic (−)-menthol and hydrophilic resorcinol was prepared and investigated as a solvent for amphiphilic caffeine. Differential scanning calorimetry and thermogravimetric analysis were first used to determine the eutectic composition and characterize its thermal behavior. The menthol-to-resorcinol molar ratio of 2:1 was subsequently selected for physicochemical investigation. Clear homogeneous solutions containing up to 0.701 mol·kg^−1^ caffeine were prepared, corresponding to 136 g of caffeine per kilogram of DES. Density, speed of sound, and viscosity were measured over the temperature range from 293.15 to 313.15 K. These data were used to evaluate the thermal expansion, volumetric and acoustic properties, intermolecular free length, and concentration and temperature dependences of viscous flow. The viscosity results were further analyzed using the Arrhenius, Jones–Dole, and Eyring–Feakins approaches and compared with the behavior of caffeine in water, ethylene glycol, and methyl salicylate. DFT calculations and molecular electrostatic potential surfaces were used to examine the local organization of the DES and the possible incorporation of caffeine through interactions with both its polar and less-polar regions. The combined experimental and computational approach provides a molecular and thermodynamic basis for evaluating this DES as a caffeine-solubilizing and delivery medium.

## 1. Introduction

Deep eutectic solvents (DESs) represent a class of liquids commonly formed by mixing a hydrogen-bond donor (HBD) and a hydrogen-bond acceptor (HBA) in an appropriate molar ratio [1]. Favorable interactions between the components can produce a pronounced melting-point depression relative to the pure constituents. DESs share several useful features with ionic liquids, while their preparation is generally simpler. Depending on the selected components, they may also exhibit lower toxicity and better biodegradability than conventional ionic liquids [2,3]. These properties have stimulated their development as alternative solvents, including applications in pharmaceutical processing, drug discovery, and drug-delivery systems [4,5].

DESs were traditionally classified into types I–IV according to the nature of the components, including quaternary ammonium salts, metal salts, and different HBDs. Type V DESs were subsequently introduced to describe nonionic systems formed exclusively from molecular components [6]. This class includes many hydrophobic DESs (HDESs), although the terms type V and hydrophobic DES are not synonymous. HDESs were first introduced in 2015 [7] and are generally composed of substances with low water solubility, such as terpenes, long-chain fatty acids or alcohols, and long-chain quaternary ammonium salts. Terpenoids, particularly menthol, are widely used in such systems because their hydrocarbon framework contributes to a nonpolar environment, while they are also renewable, commercially available, and physiologically active [6,8]. The classification of many menthol-based mixtures as DESs has been supported by melting-point depression and evidence of hydrogen bonding between the components [9,10,11]. Menthol-containing DESs have been investigated as extraction media [6,12,13], pharmaceutical crystallization media [14], solubility or permeability enhancers [15], and drug-delivery systems [16,17,18]. The permeability-enhancing, anti-inflammatory, and antimicrobial properties of menthol further support its use in the design of pharmaceutically relevant eutectic systems [19,20].

Caffeine is a widely consumed natural alkaloid with numerous applications in the food and pharmaceutical industries [21,22]. In addition to its well-known psychostimulant activity, caffeine is frequently incorporated into cosmetic and dermatological formulations intended for topical or transdermal administration [23,24]. Its topical use is associated with effects on microcirculation [25], anti-cellulite activity [26,27,28], protection against oxidative and photoinduced damage [29], and stimulation of hair growth [30,31,32]. However, the vehicle and other formulation components strongly influence caffeine solubilization, crystallization, partitioning, and penetration through the skin barrier [32,33].

New topical formulations should therefore combine efficient caffeine solubilization with suitable penetration-enhancing components. In this context, the previously unreported eutectic mixture of menthol and resorcinol is of particular interest. The polar phenolic groups of the hydrophilic resorcinol may contribute to caffeine solubilization, whereas the hydrophobic menthol provides a less polar environment and is a well-established skin-permeation enhancer. Given the therapeutic use of menthol and the application of resorcinol in dermatological products for its keratolytic and anti-inflammatory properties [34], the menthol–resorcinol system may be a useful medium for topical caffeine delivery. Direct permeation and biocompatibility studies would nevertheless be required to confirm this application.

This combination is also relevant from a molecular perspective. Caffeine is an amphiphilic molecule with an anisotropic distribution of polar and less-polar surface regions. Its carbonyl oxygen atoms and ring nitrogen atoms provide polar interaction sites, whereas its methylated regions can participate in weaker contacts with nonpolar molecular environments [35,36]. A liquid combining hydrophobic menthol and hydrophilic resorcinol may therefore provide complementary local environments for the different regions of caffeine. Such complementarity could favor caffeine incorporation through a combination of hydrogen-bonding, dipolar, and dispersive interactions and may induce reorganization of the local DES interaction network.

In this work, the solid–liquid and thermal behavior of the menthol–resorcinol system was first examined by differential scanning calorimetry over the entire composition range and by thermogravimetric analysis. Based on these results, the menthol-to-resorcinol molar ratio of 2:1 was selected for further investigation. Caffeine solutions with molalities from 0.100 to 0.701 mol·kg^−1^ were studied over the temperature range from 293.15 to 313.15 K by density, speed-of-sound, and viscosity measurements. The experimental data were used to evaluate thermal expansion, molar, partial molar, and apparent molar volumes, limiting apparent molar expansibility, isentropic compressibility, apparent molar isentropic compressibility, and intermolecular free length. The concentration and temperature dependences of viscosity were analyzed using the Arrhenius, Jones–Dole, and Eyring–Feakins approaches, and the resulting parameters were compared with those previously obtained for caffeine in water, ethylene glycol, and methyl salicylate. Finally, finite-cluster density functional theory calculations and molecular electrostatic potential analysis were used to provide qualitative insight into the local organization of the menthol–resorcinol DES and its interactions with caffeine. This combined experimental and computational approach was used to relate the molecular accommodation of caffeine to the volumetric, acoustic, and transport behavior of the system.

## 2. Results and Discussion

### 2.1. Thermal Characterization

#### 2.1.1. Differential Scanning Calorimetry (DSC) Measurements

**Figure 1:**
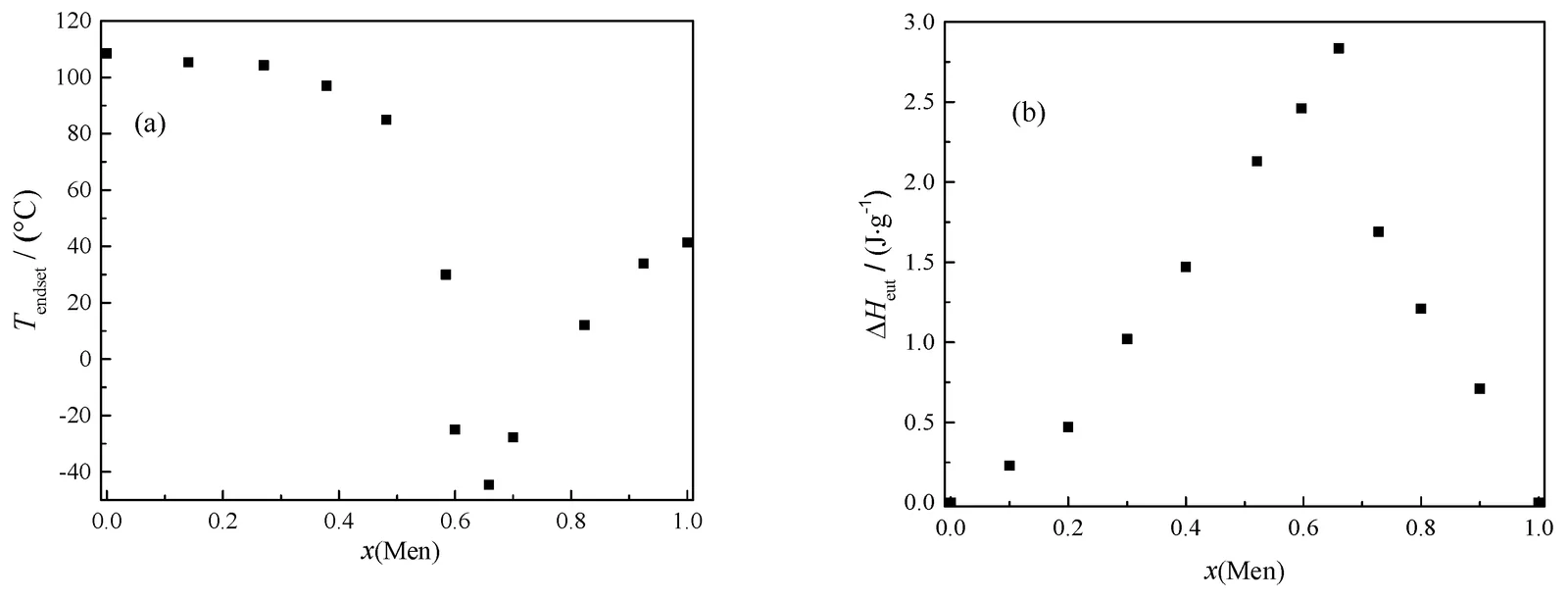
Composition dependence of (**a**) the endset temperature of the final melting transition, *T*_endset_, and (**b**) the mass–normalized enthalpy of the low–temperature eutectic melting event, Δ*H*_eut_, in the menthol–resorcinol system.

Differential scanning calorimetry was used to examine the composition-dependent melting behavior of the menthol–resorcinol system. The DSC heating curves are presented in Figure S1, while the corresponding *T*_endset_ and Δ*H*_eut_ values are listed in Table S1. Their composition dependences are shown in Figure 1a and Figure 1b, respectively.

The endset temperature of the final melting transition decreases from 108.5 °C for pure resorcinol to a minimum of −36.5 °C at *x*(Men) = 0.6667, after which it increases to 41.4 °C for pure (−)-menthol. The minimum therefore corresponds to a menthol-to-resorcinol molar ratio of 2:1. This composition was selected for the subsequent physicochemical investigation. For comparison, literature melting temperatures are approximately 109 °C for resorcinol [37] and 41–44 °C for (−)-menthol, depending on its purity and crystalline form [38].

The Δ*H*_eut_ value increases toward the same composition, reaches a maximum of 2.84 J·g^−1^ at *x*(Men) = 0.6667, and then decreases toward pure menthol. The minimum *T*_endset_ and the Δ*H*_eut_ maximum support assigning the eutectic composition near a 2:1 menthol:resorcinol mole ratio. The minimum (−36.5 °C) and the maximum (2.84 J·g^−1^) are both observed at *x*(Men) = 0.6667, providing two complementary experimental indicators that the eutectic region lies near the 2:1 menthol-to-resorcinol composition. No other screened composition exhibited a lower final melting transition. In addition, the 2:1 mixture remained homogeneous and liquid under ambient conditions and was therefore selected for subsequent physicochemical measurements.

The pronounced melting-temperature depression demonstrates strongly nonideal solid–liquid behavior. Such behavior in eutectic systems results from the combined effects of the crystal-lattice energies of the pure components, intermolecular interactions in the liquid mixture, and the entropy change accompanying mixing [39,40,41,42,43,44]. Specific interactions between the hydroxyl groups of menthol and resorcinol may contribute to the observed depression. However, the DSC results alone cannot establish the nature or geometry of these intermolecular interactions.

#### 2.1.2. Thermogravimetric (TG) Measurements

**Figure 2:**
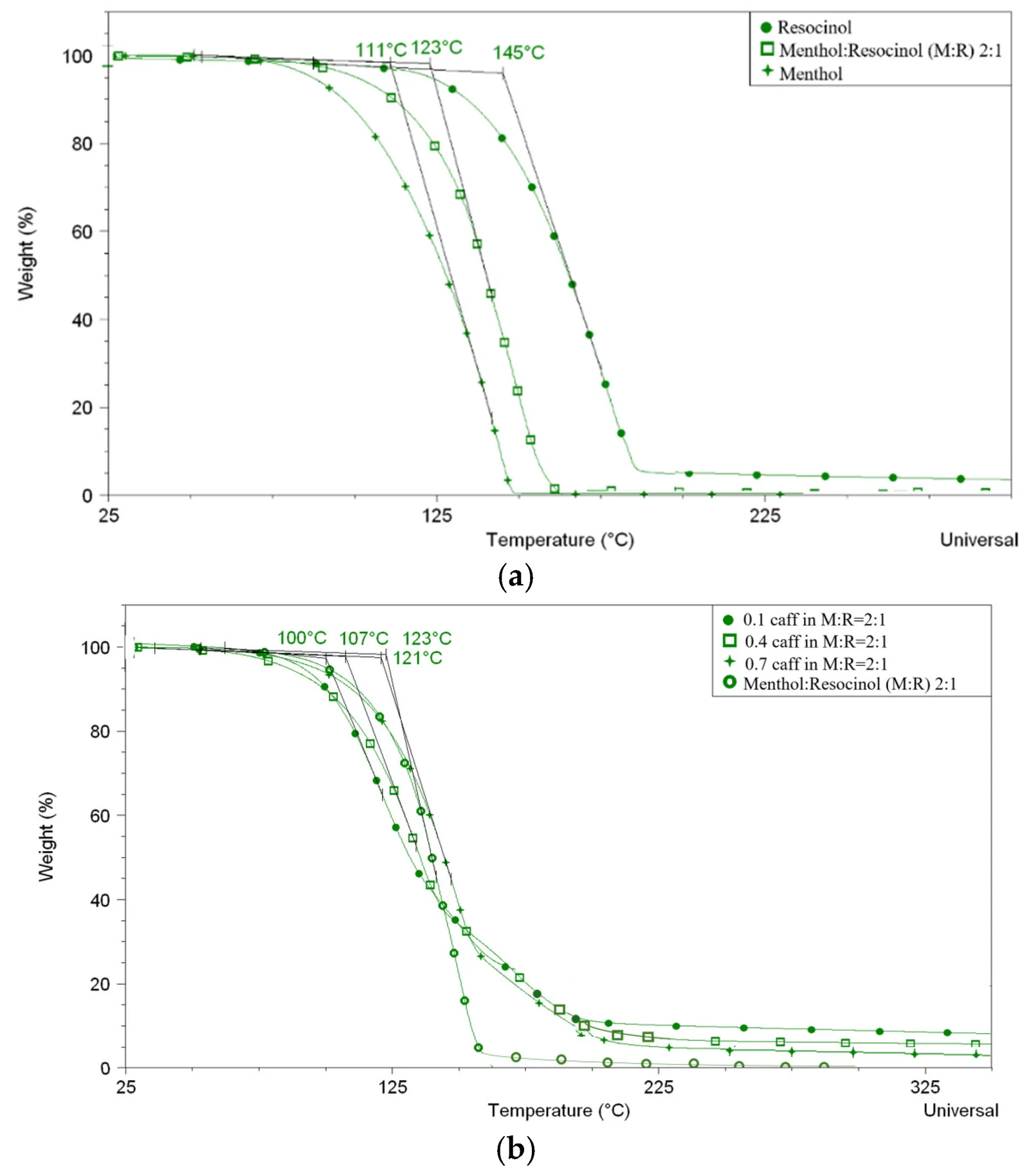
Thermogravimetric curves of (**a**) pure (−)-menthol, pure resorcinol, and the neat menthol–resorcinol DES (2:1), and (**b**) the neat DES and caffeine-containing systems at *m* = 0.100, 0.401, and 0.701 mol·kg^−1^.

Thermogravimetric analysis was used to compare the mass-loss behavior of the pure components, the neat menthol–resorcinol DES, and the caffeine-containing systems. The corresponding TG curves are presented in Figure 2, while the *T*_onset_ values are summarized in Table S2.

Pure (−)-menthol exhibits the lowest *T*_onset_ among the investigated pure components, at 111 °C, whereas the corresponding values for resorcinol and caffeine are 145 and 224 °C, respectively. The neat menthol–resorcinol DES exhibits an intermediate value of 123 °C, closer to that of menthol, consistent with its menthol-rich 2:1 composition. Because TG records mass loss arising from both volatilization and thermal decomposition, these results alone do not identify the mechanism responsible for each mass-loss step.

The caffeine-containing systems exhibit *T*_onset_ values of 100, 107, and 121 °C at *m* = 0.100, 0.401, and 0.701 mol∙kg^−1^, respectively. Thus, the initial addition of caffeine lowers the onset of mass loss relative to the neat DES, whereas *T*_onset_ progressively approaches that of the neat DES as the caffeine concentration increases. No separate mass-loss step corresponding to the decomposition of pure caffeine at approximately 224 °C is resolved in the caffeine-containing systems. Instead, their mass-loss behavior is dominated by the broad DES-related transition. This indicates that the contribution of caffeine overlaps with the mass-loss process of the DES matrix, but it does not establish the chemical mechanism of caffeine degradation or volatilization.

### 2.2. Density, Thermal Expansion, and Volumetric Properties

The experimental densities of the neat menthol–resorcinol DES and its mixtures with caffeine are reported in Table S3 of the Supporting Information. At each caffeine molality, the density decreased linearly with increasing temperature, whereas at constant temperature it increased with caffeine molality according to a second-order polynomial. These dependences are presented in Figure S2, while the corresponding fitting coefficients, standard deviations, and coefficients of determination are summarized in Tables S4 and S5.

**Figure 3:**
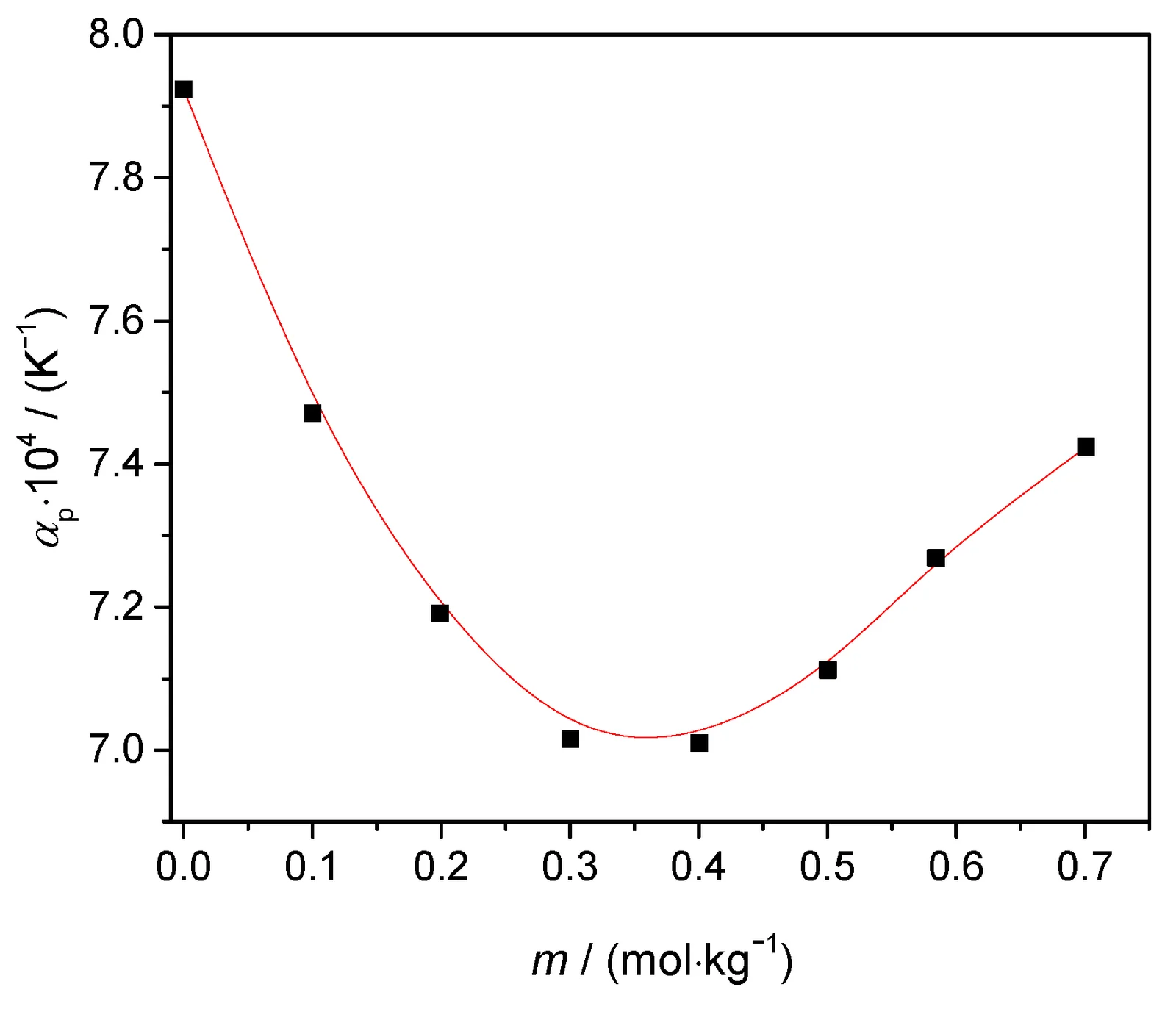
Variation in the mean isobaric thermal expansion coefficient, *α*_p_, of the neat menthol–resorcinol DES and caffeine + DES mixtures with caffeine molality in the temperature range from 293.15 to 313.15 K.

The isobaric thermal expansion coefficients, *α*_p_, calculated using Equation (3), are listed in Table S6, and their dependence on caffeine molality is shown in Figure 3. The neat DES exhibits an *α*_p_ value of 7.924 × 10^−4^ K^−1^ in the temperature range from 293.15 to 313.15 K. The addition of caffeine initially decreases *α*_p_, reaching a minimum of 7.010 × 10^−4^ K^−1^ at *m* = 0.401 mol·kg^−1^, after which the coefficient gradually increases to 7.424 × 10^−4^ K^−1^ at the highest investigated molality.

The non-monotonic dependence of *α*_p_ indicates that caffeine affects the thermal response of the DES in a concentration-dependent manner. The initial decrease in *α*_p_, reaching a minimum at *m* = 0.401 mol∙kg^−1^, is consistent with more efficient accommodation of caffeine within the DES structure and a reduced thermally induced volume expansion. At higher caffeine molalities, the gradual increase in *α*_p_ suggests a change in the structural organization of the liquid as additional caffeine is introduced. Nevertheless, the *α*_p_ values remain lower than that of the neat DES throughout the investigated concentration range, showing that caffeine overall reduces the thermal expansivity of the system.

Molar and partial molar volumes

The molar volumes, *V*_m_, of the neat DES and caffeine solutions are presented in Table S7. At each temperature, *V*_m_ decreases slightly with the initial addition of caffeine, reaches a shallow minimum near *m* = 0.4–0.5 mol·kg^−1^, and then increases slightly. The minimum occurs in the same concentration region as the minimum in *α*_p_, indicating slightly more efficient molecular packing around *m* = 0.4 mol·kg^−1^.

**Figure 4:**
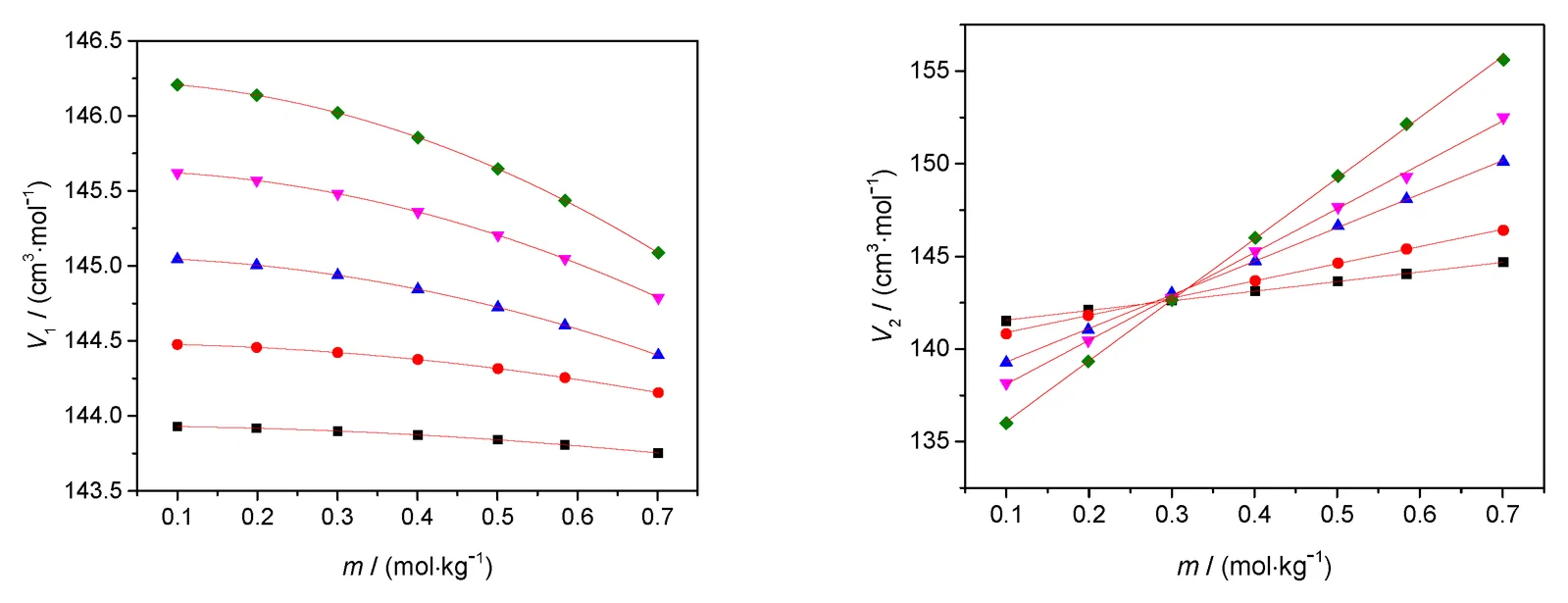
Variation in partial molar volumes for DES (**left**) and caffeine (**right**) as a function of caffeine molality, at different temperatures (*T*): = (■) 293.15; (●) 298.15; (▲) 303.15; (▼) 308.15 and (◆) 313.15 K.

**Figure 5:**
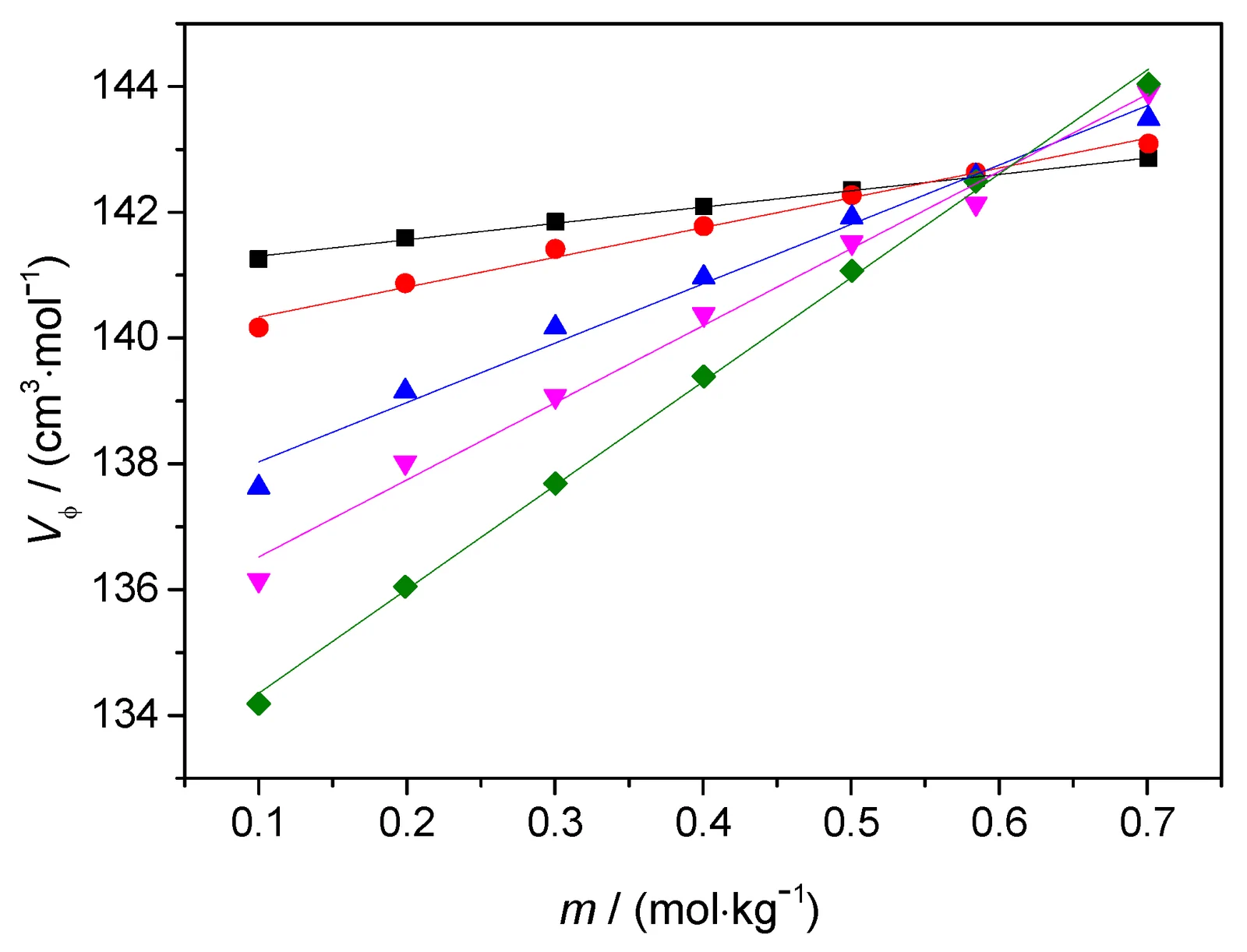
Apparent molar volume, (*V*_Φ_), as a function of (*m*) for caffeine + DES solutions at different temperatures: *T* = (■) 293.15; (●) 298.15; (▲) 303.15; (▼) 308.15 and (◆) 313.15 K. Symbols represent experimental data and solid lines correspond to Equation (8).

The apparent molar volumes of caffeine, *V*_ϕ_, and the partial molar volumes of the DES and caffeine, $\bar{V}$_1_ and $\bar{V}$_2_, respectively, were calculated using Equations (5)–(7). The resulting values are collected in Table S8, and their composition dependence is shown in Figure 4 and Figure 5.

The partial molar volume of the DES, $\bar{V}$_1_, decreases slightly as caffeine molality increases across all investigated temperatures, with a larger decrease at higher temperatures. This trend shows that caffeine changes the volumetric organization of the DES and is consistent with a slightly more compact liquid arrangement.

A more complex behavior is observed for the partial molar volume of caffeine, $\bar{V}$_2_. At caffeine molalities below approximately 0.3 mol∙kg^−1^, $\bar{V}$_2_ decreases with increasing temperature, whereas above this molality it increases with temperature, and the increase becomes more pronounced as the caffeine content rises. At lower molalities, the volumetric behavior is mainly governed by the accommodation of caffeine molecules within the DES, while at higher molalities the increasing proximity of caffeine molecules makes caffeine–caffeine interactions progressively more significant.

Apparent molar volumes

The apparent molar volumes of caffeine, *V*_ϕ_, were calculated from the experimental density data using Equation (5), and the obtained values are listed in Table S8. The *V*_ϕ_ values were then fitted as a function of caffeine molality using the Masson equation modified for nonelectrolyte solutions [45], Equation (8). The corresponding linear fits are presented in Figure 5, while the fitting parameters and statistical indicators are summarized in Table 1.

The parameter *S*_v_ from the Masson equation describes the concentration dependence of the apparent molar volume and is commonly used as an indicator of the net contribution of solute–solute interactions. The *S*_v_ values for caffeine in the menthol–resorcinol DES are positive throughout the investigated temperature range and increase markedly from 2.60 cm^3^·kg·mol^−2^ at 293.15 K to 16.5 cm^3^·kg·mol^−2^ at 313.15 K (Table 1).

**Figure 6:**
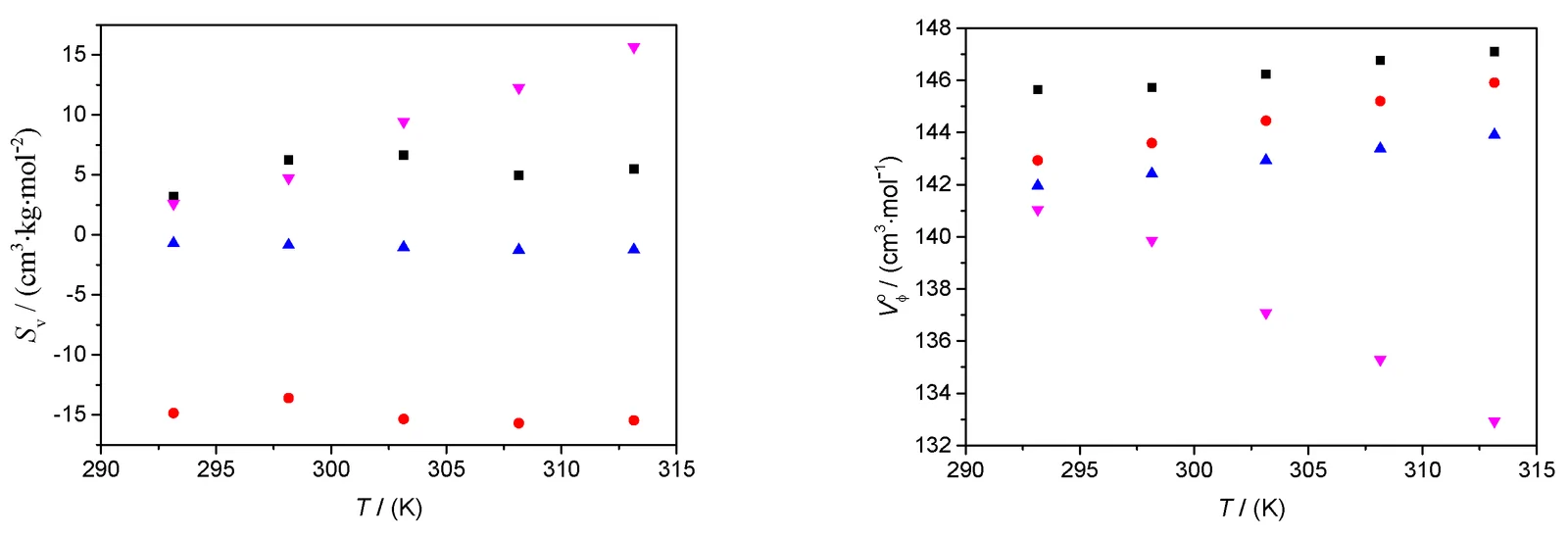
Variation in the *S*_v_ coefficient values (**left**) and apparent molar volume (**right**) of caffeine at infinite dilution ($V_{\phi }^{o}$) with temperature in: ethylene glycol (■), water (●), methyl salicylate (▲) and menthol–resorcinol eutectic solvent (▼). Literature data for water, ethylene glycol, and methyl salicylate are from refs. [46,47,48].

To evaluate the effect of the solvent environment on caffeine–caffeine interactions, the temperature dependence of *S*_v_ for caffeine in the menthol–resorcinol DES was compared with the corresponding values previously reported in water [46], ethylene glycol [47], and methyl salicylate [48] (Figure 6).

The *S*_v_ values are negative in water and methyl salicylate, whereas they are positive in ethylene glycol and in the menthol–resorcinol DES. In aqueous solutions, the negative values were associated with weak caffeine–caffeine interactions, and molecular dynamics simulations showed that caffeine molecules remained dispersed below the solubility limit [46]. In methyl salicylate, the negative *S*_v_ values and molecular dynamics simulations likewise confirmed the absence of caffeine self-aggregates, indicating effective solvation of caffeine by this solvent [48]. In contrast, the positive *S*_v_ values obtained in ethylene glycol were associated with pronounced caffeine self-association. Molecular dynamics simulations showed a very low caffeine solvation number in ethylene glycol compared with water and confirmed the formation of caffeine aggregates [47]. These results indicated that self-association in ethylene glycol arises primarily from weak caffeine-solvent interactions and poor solvation, rather than from unusually strong direct attraction between caffeine molecules. At 293.15 and 298.15 K, the *S*_v_ values in the DES are comparable to or slightly lower than those in ethylene glycol, whereas above 303.15 K they become considerably higher. The strong increase in *S*_v_ with temperature indicates that caffeine–caffeine interactions become progressively more pronounced upon heating.

Together with the change in the temperature dependence of $\bar{V}$_2_ observed around *m* = 0.3 mol·kg^−1^, the positive and increasing *S*_v_ values suggest that caffeine–caffeine contacts become increasingly important above this molality. A possible explanation is that, with increasing temperature and caffeine concentration, the DES components become less effective at maintaining separate solvation environments around individual caffeine molecules, which increases the tendency toward caffeine self-association. However, the *S*_v_ values alone do not prove aggregate formation, and this interpretation should be verified by molecular dynamics simulations or spectroscopic measurements.

The second parameter obtained from the Masson equation is the limiting apparent molar volume, *V*_ϕ_^o^. At infinite dilution, *V*_ϕ_^o^ is equal to the limiting partial molar volume of caffeine and represents the change in solution volume caused by the addition of one mole of caffeine to an effectively infinite amount of solvent, where caffeine–caffeine interactions are negligible. The obtained values are listed in Table 1 and compared with those previously reported for caffeine in water [46], ethylene glycol [47], and methyl salicylate [48] in Figure 6.

The *V*_ϕ_^o^ values for caffeine in the menthol–resorcinol DES are lower than those reported for water, ethylene glycol, and methyl salicylate throughout the investigated temperature range. Moreover, the DES is the only solvent in which *V*_ϕ_^o^ decreases with temperature, from 141.04 cm^3^·mol^−1^ at 293.15 K to 132.70 cm^3^·mol^−1^ at 313.15 K, whereas it increases upon heating in the other solvents [46,47,48]. Because *V*_ϕ_^o^ refers to infinite dilution, this behavior cannot be attributed to caffeine–caffeine interactions and instead reflects the local response of the solvent environment to an isolated caffeine molecule. The opposite temperature dependence observed in the menthol–resorcinol DES therefore indicates a distinctly different mechanism of caffeine accommodation, in which the volumetric contribution of caffeine becomes progressively smaller as the temperature increases.

The menthol–resorcinol DES is a hydrogen-bonded liquid whose density decreases and molar volume increases with temperature. Heating increases molecular mobility and weakens part of the intermolecular organization of non-ionic menthol-based DESs, thereby increasing the dynamically available intermolecular space within the liquid [49,50]. Under these conditions, adding a caffeine molecule requires progressively less additional expansion of the DES. Caffeine can be accommodated within the thermally generated intermolecular space while inducing a local rearrangement of the surrounding menthol and resorcinol molecules. Consequently, the effective contribution of one mole of caffeine to the total solution volume at infinite dilution becomes smaller as the temperature increases, which is reflected in the decrease in *V*_ϕ_^o^.

Thus, the lower and decreasing *V*_ϕ_^o^ values should not be interpreted solely as evidence of weaker caffeine–DES interactions. They may reflect the combined effects of caffeine–DES interactions, solvent reorganization, and more efficient packing of isolated caffeine molecules within the DES structure. Therefore, the magnitude of *V*_ϕ_^o^ alone cannot be used to rank the strength of caffeine–solvent interactions in different solvents.

The temperature dependence of *V*_ϕ_^o^ was described by the second-order polynomial given in Equation (9), and the corresponding fitting coefficients are reported in Table S9. Differentiation according to Equation (10) yielded the limiting apparent molar expansibility, *E*_ϕ_^o^, whose values are listed in Table S10.

**Figure 7:**
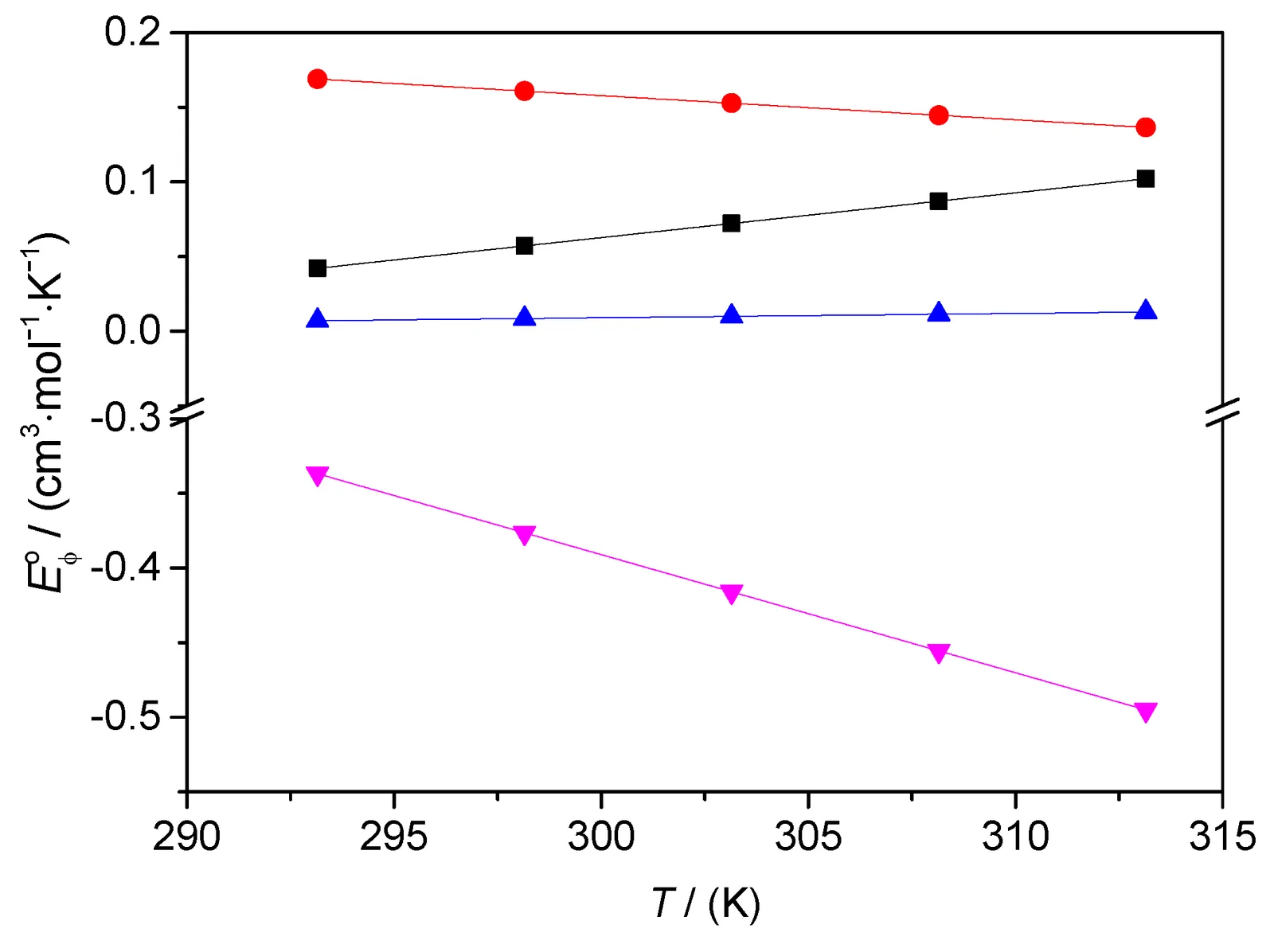
Variation in the limiting apparent molar expansibility, (*E*_ϕ_^o^) of caffeine in (●) water, (■) ethylene glycol, (▲) methyl salicylate and (▼) menthol–resorcinol eutectic solvent with temperature.

For caffeine in the menthol–resorcinol DES, *E*_ϕ_^o^ is negative at all investigated temperatures and decreases from −0.304 to −0.515 cm^3^·mol^−1^·K^−1^. In contrast, positive values were obtained for caffeine in water, ethylene glycol, and methyl salicylate (Figure 7) [46,47,48]. The negative *E*_ϕ_^o^ values confirm that the volumetric contribution of an isolated caffeine molecule decreases as the temperature rises, demonstrating a distinctly different temperature response of caffeine solvation in the DES. The negative *E*_ϕ_^o^ values have a direct volumetric meaning. Although the neat menthol–resorcinol DES expands upon heating, the limiting apparent molar volume of caffeine decreases with temperature. Thus, at infinite dilution, caffeine makes a negative contribution to the thermal expansibility of the system and partially counteracts the volume expansion of the neat DES. In other words, the addition of caffeine at infinite dilution produces a progressively smaller increase in solution volume as the temperature rises. This behavior differs from that observed in water, ethylene glycol, and methyl salicylate, where positive *E*_ϕ_^o^ values indicate that the limiting volumetric contribution of caffeine increases upon heating. The corresponding second derivative, (∂*E*_ϕ_^o^/∂*T*)*_p_* = (∂^2^*V*_ϕ_^o^/∂*T*^2^)*_p_* = 2*a*_2_, is approximately −1.05 × 10^−2^ cm^3^·mol^−1^·K^−2^. In aqueous solutions, a negative value of this parameter, commonly referred to as the Hepler coefficient [51], is frequently associated with structure-breaking behavior of the solute. However, this terminology should be applied cautiously to a nonaqueous, multicomponent DES. In the present system, the negative Hepler coefficient is more appropriately interpreted as evidence that the local organization of the DES around caffeine at infinite dilution becomes increasingly responsive to temperature, resulting in a progressively smaller volumetric contribution of caffeine as the temperature rises.

### 2.3. Acoustic Properties

The experimental speeds of sound, *u*, together with the corresponding density data, were used to calculate the isentropic compressibilities, *κ*_s_, according to Equation (11). The obtained *u* and *κ*_s_ values are listed in Table S11. Over the investigated temperature range, the speed of sound decreases by approximately 8.0–9.3% upon heating, depending on caffeine molality. At all investigated temperatures, the speed of sound generally increases with caffeine molality. Since the speed of sound depends simultaneously on density and compressibility, the interpretation of acoustic behavior is based primarily on the derived *κ*_s_ values.

The isentropic compressibility increases by approximately 20–23% between 293.15 and 313.15 K for the pure DES and all caffeine-containing systems (Table S11), showing that the liquids become more compressible upon heating. In contrast, caffeine addition decreases *κ_s_* at every temperature. At the highest investigated molality, the reduction relative to the pure DES is approximately 8.2% at 293.15 K and 5.4% at 313.15 K. Thus, although heating increases the magnitude of pressure-induced volume fluctuations, caffeine partially suppresses this effect and produces a less compressible liquid arrangement. This behavior is consistent with the lower thermal expansivity observed for the caffeine-containing systems.

The acoustically derived intermolecular free length, *L*_f_, was calculated using Equations (12) and (13), and the resulting values are summarized in Table 2. Consistent with the increase in *κ_s_*, *L*_f_ increases by approximately 13.7–15.2% over the investigated temperature interval. At every temperature, caffeine addition decreases *L*_f_, with the reduction at the highest caffeine molality amounting to approximately 4.0% at 293.15 K and 2.8% at 313.15 K. These results indicate that heating increases the dynamically available intermolecular space within the DES, whereas caffeine partially occupies this space and reduces the overall compressibility of the liquid. The increase in *L*_f_ for the pure DES also provides acoustic support for the temperature-dependent caffeine accommodation proposed from the decrease in *V*_ϕ_^o^. Because the Jacobson relation is empirical, *L*_f_ should be regarded as an acoustic indicator of dynamic intermolecular separation rather than as a direct geometrical measure of permanent molecular cavities.

The apparent molar isentropic compressibilities of caffeine, *κ*_ϕ_, were calculated using Equation (14). These values are listed in Table S12. The *κ*_ϕ_ values are positive throughout the investigated concentration range and increase with temperature at every caffeine molality. This shows that the apparent molar contribution of caffeine to compressibility becomes larger as the investigated system is heated.

**Figure 8:**
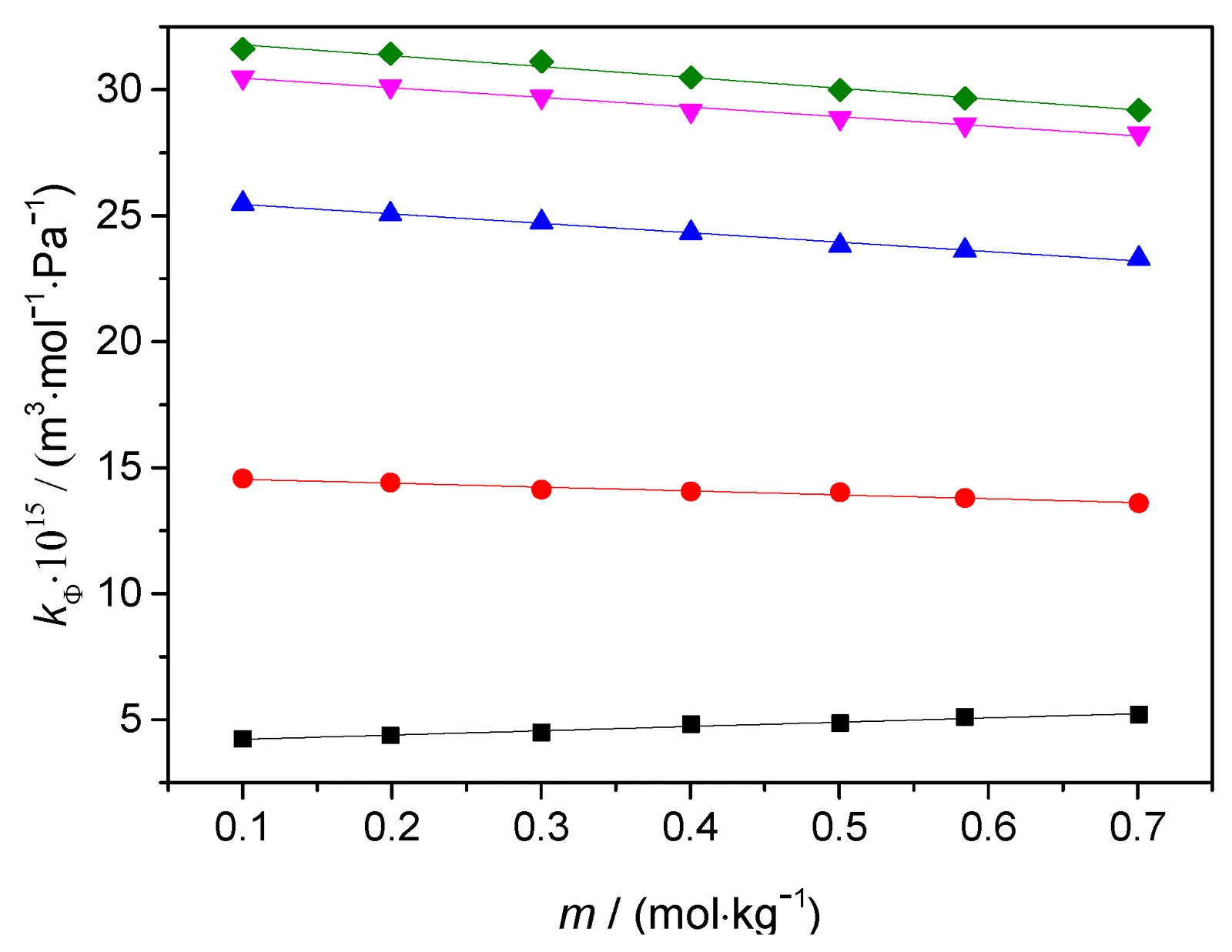
Apparent molar isentropic compressibility, *κ*_ϕ_, of caffeine in DES as a function of molality at different temperatures: (*T*): = (■) 293.15; (●) 298.15; (▲) 303.15; (▼) 308.15; (◆) 313.15 K.

At each temperature, the dependence of *κ*_ϕ_ on caffeine molality was described by Equation (15). The experimental values and linear fits are presented in Figure 8, while the limiting apparent molar isentropic compressibilities, *κ*_ϕ_^o^, and the concentration coefficients, *S*_κ_, are summarized in Table 3. The parameter *κ*_ϕ_^o^ represents the extrapolated apparent molar isentropic compressibility at infinite dilution, whereas *S*_κ_ describes its change with caffeine molality. At 293.15 K, *κ*_ϕ_ increases slightly with molality and *S*_κ_ is positive. From 298.15 to 313.15 K, *κ*_ϕ_ decreases with molality and *S*_κ_ is negative.

From *T* = 298.15 K, the negative *S*_κ_ values show that the apparent molar compressibility contribution of caffeine becomes smaller as its concentration increases. This agrees with the decrease in the bulk isentropic compressibility, *κ_s_*, and the intermolecular free length, *L_f_*, upon caffeine addition (Table S11 and Table 2). Therefore, increasing caffeine molality makes the bulk liquid less compressible and reduces the acoustically derived intermolecular free space. A similar combination of positive *κ*_ϕ_ values and decreasing bulk *κ*_s_ with caffeine concentration was previously observed in methyl salicylate [48]. These quantities are not contradictory because *κ*_ϕ_ describes the molar contribution of caffeine relative to the pure solvent, whereas *κ*_s_ describes the compressibility of the entire solution.

The acoustic results are also consistent with the volumetric analysis. The positive Masson *S*_v_ values show that concentration-dependent contributions to the apparent molar volume become more pronounced as the caffeine content increases. The negative *S*_κ_ values show that the corresponding concentration-dependent arrangements have a lower apparent molar compressibility than caffeine at infinite dilution. Together with the change in the temperature dependence of the partial molar volume of caffeine above approximately 0.3 mol·kg^−1^, these results indicate that caffeine–caffeine contacts and collective rearrangement of the DES become increasingly important at higher caffeine concentrations. These arrangements increase the concentration-dependent volumetric contribution but reduce the system’s response to pressure-induced volume changes.

### 2.4. Viscosity of Caffeine + DES Systems

The experimental dynamic viscosities of the neat menthol–resorcinol DES and the caffeine-containing systems are listed in Table S13. At every composition, viscosity decreases strongly with increasing temperature. The effect of caffeine concentration is non-monotonic in the low-molality region. At *m* = 0.100 mol·kg^−1^, viscosity decreases slightly at most temperatures, with the largest reduction of approximately 11% observed at 298.15 K. At higher caffeine molalities, viscosity increases continuously. At the highest investigated molality, *m* = 0.701 mol·kg^−1^, viscosity is 110.4% higher than that of the pure DES at 293.15 K and 69.8% higher at 313.15 K.

The shallow viscosity minimum at low caffeine content indicates that the initial addition of caffeine does not increase flow resistance and may slightly facilitate molecular rearrangement within the DES. This low-concentration viscosity minimum is qualitatively consistent with the limiting volumetric results, which indicate that caffeine can be accommodated within the DES with limited additional expansion of the solvent structure. Above approximately 0.1 mol·kg^−1^, further caffeine addition progressively restricts molecular rearrangement and increases resistance to viscous flow. This trend agrees with the decrease in isentropic compressibility and intermolecular free length and with the increasing contribution of caffeine–caffeine contacts indicated by the positive Masson *S*_v_ values.

Newtonian behavior was examined at *T* = 298.15 K for the pure DES and for the solution containing *m* = 0.597 mol∙kg^−1^ caffeine. In both systems, viscosity remained constant over the investigated shear-rate range, while shear stress increased linearly with shear rate, confirming Newtonian flow behavior. The corresponding data and plots are presented in Table S14 and Figure S3 of the Supporting Information.

The temperature dependence of viscosity was analyzed using the Arrhenius equation given by Equation (16). The corresponding linear dependences of ln*η* on 1/*T* are presented in Figure S4 of the Supporting Information, while the calculated values of the pre-exponential factor, *η*_A_, and the apparent activation energy of viscous flow, *E*_η_, are summarized in Table S15. The Arrhenius equation satisfactorily describes the experimental data at all investigated caffeine molalities, with *R*^2^ values ranging from 0.9961 to 0.9990. The *E*_η_ value shows a shallow minimum, decreasing from approximately 111.0 kJ·mol^−1^ for the neat DES to 108.7 kJ·mol^−1^ at *m* = 0.100 mol∙kg^−1^, in agreement with the small viscosity decrease observed after the initial addition of caffeine. At higher molalities, *E*_η_ increases continuously and reaches approximately 118.1 kJ·mol^−1^ at *m* = 0.701 mol∙kg^−1^, indicating that molecular rearrangements required for viscous flow become progressively more energetically demanding as the caffeine content increases.

The concentration dependence of viscosity is commonly described using the Jones–Dole equation [52], given for the present nonelectrolyte systems by Equation (18). The quantity *η*/*η*_0_ − 1 represents the specific viscosity, *η_sp_*. Its linear dependence on caffeine molar concentration is presented in Figure S5, and the viscosity *B*-coefficient is obtained from the slope of this dependence. The resulting values are summarized in Table 4.

The Jones–Dole *B*-coefficient has several physicochemical implications because it reflects the combined effects of solute size, solute–solvent interactions, and solvent reorganization on viscous flow [53]. One of its most important applications is the qualitative assessment of the influence of a solute on the structural organization of the solvent. In aqueous solutions, positive *B* values that decrease with increasing temperature are commonly associated with structure-making behavior, whereas negative values are regarded as structure-breaking properties [54].

The *B*-coefficients for caffeine in the menthol–resorcinol DES are positive throughout the investigated temperature range and decrease from 2.122 dm^3^·mol^−1^ at 293.15 K to 1.308 dm^3^·mol^−1^ at 313.15 K. According to the conventional Jones–Dole criterion, caffeine therefore behaves as a classical structure maker in menthol–resorcinol DES. The positive values indicate that caffeine increases the resistance to viscous flow and promotes a more strongly organized local environment, whereas their decrease with temperature shows that this effect weakens as thermal motion makes the hydrogen-bonded DES structure more labile.

Comparison with other solvents shows that the *B*-coefficients in the DES are by far the largest over the entire temperature range (Table 4). In ethylene glycol, the values are negative and become slightly more negative as temperature increases, indicating that caffeine is a structure breaker in this solvent [47]. In water, the coefficients are positive and increase slightly with temperature, consistent with the previously described atypical structure-making behavior of caffeine [46]. Positive values that decrease with temperature are also obtained in methyl salicylate [48], but they are markedly smaller than those in the DES.

An important question therefore arises. The analogous menthol–thymol hydrophobic type V DES is already a highly associated liquid with an extensive hydrogen-bond network [50,55,56,57]. Replacing thymol with resorcinol introduces an additional hydroxyl group and increases the number of possible hydrogen-bonding configurations. How, then, can caffeine further increase the organization of an already highly associated DES, as suggested by its exceptionally high positive B-coefficients?

The first and most conservative explanation concerns the applicability of the structure-making/structure-breaking concept itself. This interpretation of the Jones–Dole B-coefficient was developed primarily for aqueous solutions, and its direct transfer to nonaqueous, multicomponent liquids is not necessarily straightforward. Consequently, the positive *B*-coefficients and their decrease with temperature may primarily describe the strong increase in resistance to viscous flow caused by caffeine, rather than provide unequivocal evidence of increased structural order [58].

A complementary molecular explanation can be considered based on the amphiphilic character of caffeine [35,36]. Its carbonyl oxygen atoms serve as strong hydrogen-bond acceptors, whereas the methylated regions and other less polar molecular surfaces can engage in weaker dispersive contacts. Caffeine can therefore interact with the chemically heterogeneous environment provided by the hydroxyl groups and hydrocarbon regions of the menthol–resorcinol DES. The DFT results discussed in Section 2.5 provide a qualitative illustration of how caffeine can be incorporated into the local DES interaction network without completely disrupting the principal menthol–resorcinol hydrogen bonds. Such local reorganization provides a plausible molecular interpretation of the large positive B-coefficients and is qualitatively consistent with the decrease in intermolecular free length and isentropic compressibility.

Activation parameters of viscous flow

To further examine how caffeine affects the molecular rearrangements required for viscous flow, the corresponding activation parameters were evaluated using the Eyring–Feakins approach. All activation parameters discussed below were calculated using Equations (19)–(22). Equation (19) was used to calculate the standard molar Gibbs energy of activation for viscous flow of the pure DES, $\Delta \mu_{1}^{o\neq }$, whereas Equation (20) was used to calculate the corresponding quantity associated with caffeine at infinite dilution, $\Delta \mu_{2}^{o\neq }$. The entropy and enthalpy of activation associated with caffeine, $\Delta S_{2}^{o\neq }$ and $\Delta H_{2}^{o\neq }$, were then obtained using Equations (21) and (22), respectively. The calculated values are given in Table S16 and Table 5 and provide an additional thermodynamic basis for evaluating the effect of caffeine on viscous flow in the menthol–resorcinol DES.

At all investigated temperatures, $\Delta \mu_{2}^{o\neq }$ is higher than $\Delta \mu_{1}^{o\neq }$, indicating that caffeine increases the Gibbs energy of activation for viscous flow relative to the pure DES. The difference decreases from 35.9 kJ·mol^−1^ at 293.15 K to 23.0 kJ·mol^−1^ at 313.15 K, indicating that this contribution weakens with increasing temperature, consistent with the decrease in the *B*-coefficient in the Jones-Dole equation. The opposite behavior was reported for caffeine in ethylene glycol, where $\Delta \mu_{2}^{o\neq }$ < $\Delta \mu_{1}^{o\neq }$, consistent with the negative *B*-coefficients and the viscosity-lowering effect of caffeine [47].

The temperature dependence of $\Delta \mu_{2}^{o\neq }$ (Equations (21) and (22)) gives positive values of approximately $\Delta H_{2}^{o\neq }$ = 325 kJ·mol^−1^ and $\Delta S_{2}^{o\neq }$ = 875 J·mol^−1^·K^−1^ (Table 5). These values indicate that the formation of the activated state is accompanied by weakening and reorganization of intermolecular interactions, and an increase in disorder. Positive values of $\Delta H_{2}^{o\neq }$ were also obtained for caffeine in ethylene glycol, water, and methyl salicylate (Table 5). However, the value obtained in the menthol–resorcinol DES is several times higher, indicating a substantially greater enthalpic requirement for formation of the activated state. Positive values of $\Delta S_{2}^{o\neq }$ were also obtained in ethylene glycol and methyl salicylate, showing that formation of the activated state is accompanied by an increase in disorder. This effect is considerably more pronounced in the investigated DES. In contrast, the negative activation entropy obtained in water indicates that the activated state is more ordered and configurationally more restricted than the equilibrium state. These differences likely reflect the combined effects of solvent molecular size, shape, and symmetry, the strength and directionality of solvent–solvent and caffeine–solvent interactions, and the geometrical and packing changes required to form the activated state for viscous flow.

### 2.5. DFT Analysis of Caffeine–DES Interactions

**Figure 9:**
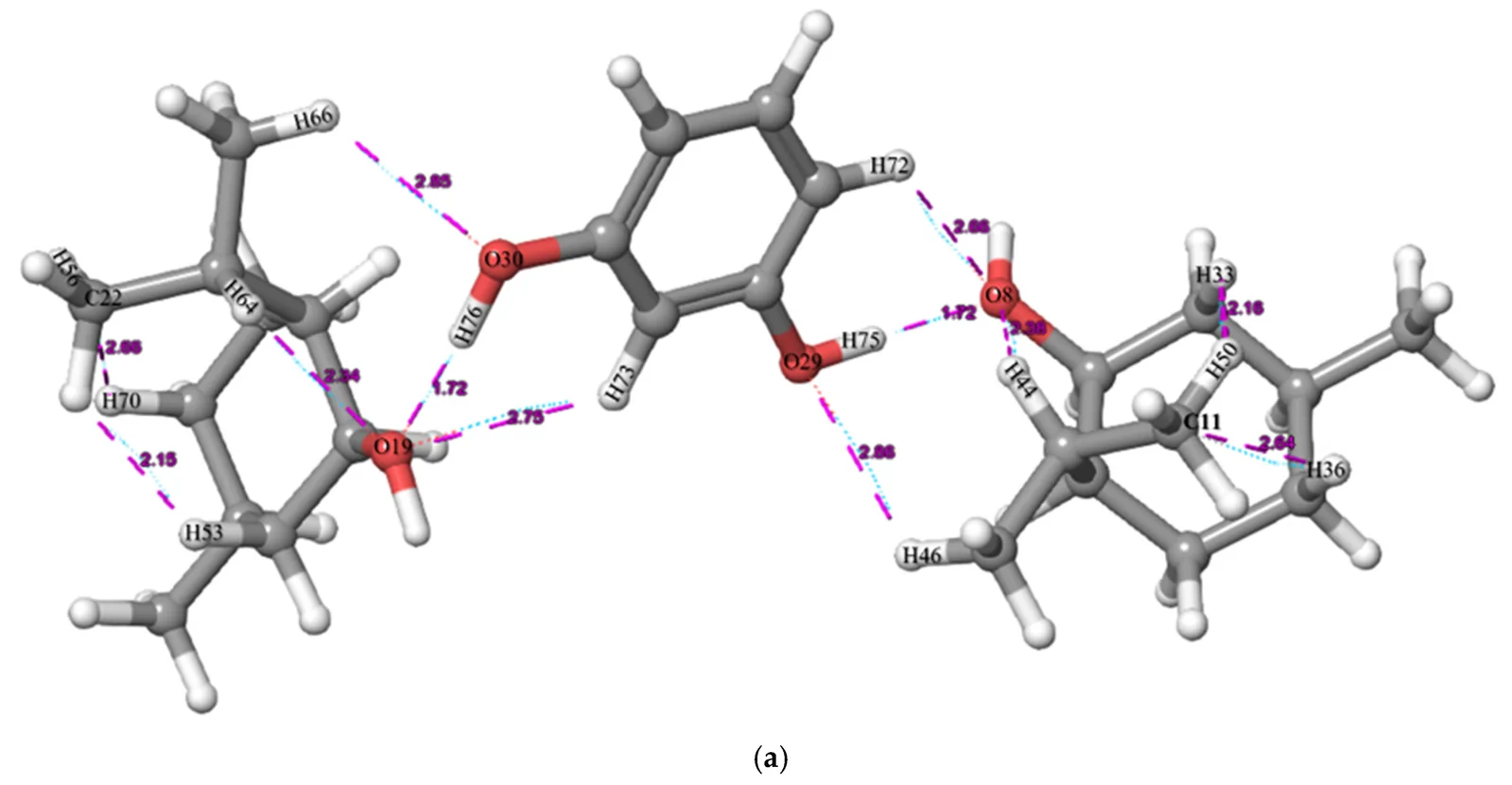

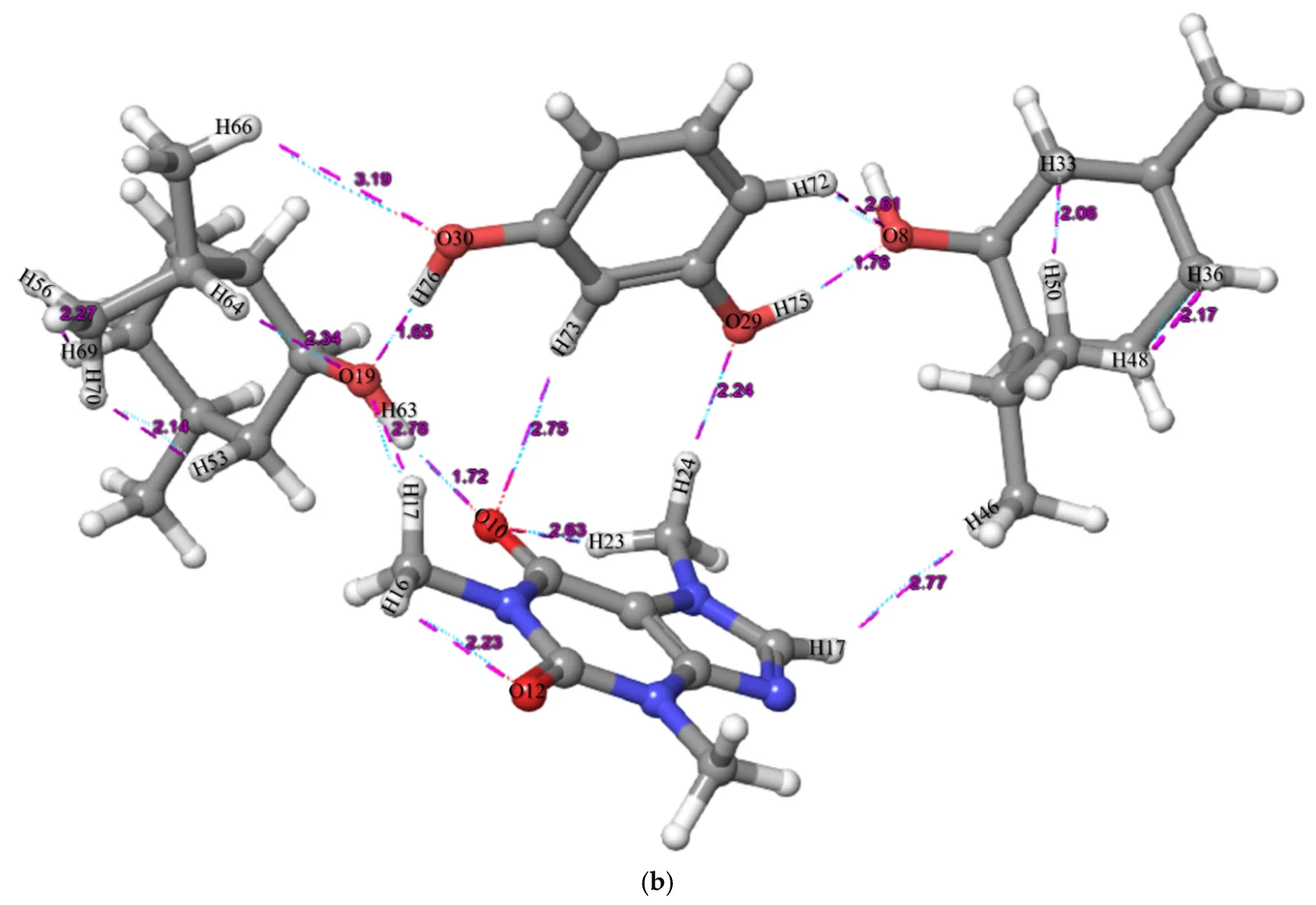
DFT-optimized structures of the representative molecular clusters containing (**a**) two menthol molecules and one resorcinol molecule and (**b**) the same menthol–resorcinol cluster with one caffeine molecule. Dashed lines indicate selected intermolecular contacts, and distances are given in Å.

DFT calculations were performed on representative finite clusters to provide qualitative molecular-level support for interpreting the experimental results. The first cluster contained two menthol molecules and one resorcinol molecule, corresponding to the 2:1 molar composition of the neat DES. The second cluster contained the same DES components together with one caffeine molecule. Their optimized structures are presented in Figure 9, while the selected intermolecular distances are summarized in Table S17.

In the optimized DES cluster, the resorcinol molecule is located between the two menthol molecules. Its two phenolic OH groups form hydrogen bonds with the oxygen atoms of the menthol molecules, with H76∙∙O19 and H75∙∙O8 distances of 1.72 Å. This representative arrangement illustrates how resorcinol can connect two menthol molecules through simultaneous heteromolecular hydrogen bonds and provides qualitative molecular support for the formation of the nonionic type V DES. These heteromolecular O–H···O hydrogen bonds favor menthol–resorcinol contacts over the self-association patterns present in the pure crystalline components and can interfere with the regular molecular packing required for crystallization. Stabilization of mixed liquid configurations, together with the entropy of mixing, lowers the chemical potential of the liquid relative to the separate solid phases, thereby contributing to the observed melting-point depression. The finite-cluster calculation does not reproduce the phase equilibrium and cannot quantify the melting-point depression. Instead, it provides a representative local interaction motif that is consistent with the DSC results.

After caffeine addition, the two principal menthol–resorcinol hydrogen bonds are retained, although their distances change to 1.65 and 1.76 Å, indicating an asymmetric local reorganization of the original cluster. At the same time, an additional O–H ∙∙∙ O=C interaction is formed between the hydroxyl group of menthol and one carbonyl oxygen atom of caffeine, with an H63 ∙∙∙ O10 distance of 1.72 Å. A weaker C–H ∙∙∙ O contact between caffeine and resorcinol is also observed, with an H24 ∙∙∙ O29 distance of 2.24 Å. Thus, in this representative cluster, caffeine becomes incorporated into the local interaction network without complete disruption of the two principal menthol–resorcinol hydrogen bonds.

The MEP surfaces provide additional insight into the electrostatic complementarity of the components (Figures S6–S8). The DES cluster exhibits a heterogeneous electrostatic surface, with strongly polar regions around the hydroxyl groups and less-polar regions associated mainly with the hydrocarbon portions of menthol. Caffeine shows a similarly anisotropic surface: its carbonyl oxygen atoms are the most electron-rich sites, whereas its methylated regions are less polar. This combination may allow caffeine to participate in hydrogen-bonding interactions while its less-polar regions are accommodated close to the hydrocarbon environment of menthol through weaker dispersive contacts. This interpretation is consistent with the amphiphilic character of caffeine [35,36] and with the optimized DES–caffeine cluster, in which a menthol hydroxyl group forms an O–H···O=C interaction with caffeine.

Retention of the principal menthol–resorcinol hydrogen bonds together with the formation of an additional caffeine–menthol interaction provides a plausible molecular basis for the experimentally observed increase in resistance to viscous flow at higher caffeine contents. Incorporation of caffeine into an interaction network involving both DES components may require a more cooperative molecular rearrangement during flow. This interpretation is qualitatively consistent with the large positive viscosity B-coefficients, the decrease in intermolecular free length and isentropic compressibility, and the comparatively large activation enthalpy and entropy of viscous flow.

## 3. Materials and Methods

### 3.1. Materials

(−)-Menthol, resorcinol, and caffeine were of analytical grade and were used as received without further purification. Their provenance and main specifications are summarized in Table 6. All measurements were performed at atmospheric pressure, *p* = 1 × 10^5^ Pa.

### 3.2. Preparation of DES and Caffeine Solutions

Menthol–resorcinol mixtures were prepared gravimetrically in sealed glass vials. A composition screening series was prepared over the complete range from *x*(Men) = 0 to *x*(Men) = 1 in mole fraction increments of 0.1, with the pure components serving as the endpoint compositions. The binary mixtures were mechanically agitated at approximately 80 °C for 30 min until clear homogeneous phases were obtained. An additional mixture with a menthol-to-resorcinol molar ratio of 2:1, corresponding to *x*(Men) = 0.667, was prepared for detailed characterization. After preparation, the samples were cooled to room temperature and visually inspected. The selected 2:1 composition remained a transparent, single-phase liquid after 24 h under ambient conditions and was stored in tightly closed vials prior to further measurements.

Caffeine solutions in the selected 2:1 menthol–resorcinol DES were prepared gravimetrically using a Mettler Toledo analytical balance with a readability of 0.1 mg. The samples were heated to 80 °C under mechanical stirring until caffeine dissolved completely and clear homogeneous liquid phases were obtained. The investigated caffeine molalities ranged from 0.100 to 0.701 mol∙kg^−1^. The standard uncertainty in molality was below 5 × 10^−4^ mol∙kg^−1^. It should be noted that the highest investigated molality, *m* = 0.701 mol∙kg^−1^ corresponds to 136.1 g of caffeine per 1000 g of DES. The solution remained clear and homogeneous at *T* = 293.15 K, and this concentration is approximately 8.3 times the reported solubility of caffeine in water at the same temperature [46].

### 3.3. Apparatus and Procedure

#### 3.3.1. Differential Scanning Calorimetry

Differential scanning calorimetry measurements were performed using a Mettler Toledo DSC1 instrument (Greifensee, Switzerland). Between 6 and 10 mg of each sample was weighed into a 40 μL aluminum crucible and hermetically sealed. An empty sealed crucible was used as the reference. Each sample was cooled from 25 to −70 °C at 2 °C·min^−1^, held at −70 °C for 5 min, and subsequently heated to 150 °C at 2 °C·min^−1^ under a nitrogen flow of 50 mL·min^−1^. Temperature and enthalpy calibrations were performed using indium and zinc standards supplied by Mettler Toledo. DSC endset temperatures and normalized melting enthalpies were determined from the heating scans using the accompanying STARe Eval software. The estimated standard uncertainty of the reported transition temperatures was 0.5 °C. The endset temperature of the final melting transition, *T*_endset_, was used as an experimental estimate of the liquidus temperature. For the binary mixtures, the low-temperature eutectic endotherm was integrated using the same baseline procedure and normalized to the sample mass. The resulting mass-normalized enthalpy is denoted as Δ*H*_eut_ and reported in J·g^−1^. Enthalpy values are reported as positive values according to the thermodynamic convention for melting. For the pure components, Δ*H*_eut_ was set to zero because the eutectic melting event is absent; these values do not represent their total enthalpies of fusion. The DSC composition screening was used to identify the composition with the lowest endset temperature and to select the menthol-to-resorcinol molar ratio of approximately 2:1 for subsequent measurements.

#### 3.3.2. Thermogravimetric Analysis

Thermogravimetric measurements were performed using an SDT Q600 simultaneous thermal analyzer (TA Instruments, New Castle, Delaware, USA). Approximately 3 mg of each pure compound, the pure DES, or caffeine-containing DES solution was placed in an open platinum pan. The samples were heated from room temperature to 500 °C at 10 °C·min^−1^ under an argon purge of 100 cm^3^·min^−1^. Temperature calibration over the range 25–1000 °C was performed using the Curie point of a nickel standard. The onset temperature of mass loss, *T*_onset,_ was determined using the tangent-intersection method. The thermograms were evaluated using TA Instruments Universal Analysis 2000 software (version 4.5A, Build 4.5.0.5).

#### 3.3.3. Density and Sound Velocity Measurements

Density and speed of sound were measured using an Anton Paar DSA 5000 M vibrating-tube density (Graz, Austria) and sound-velocity analyzer. Measurements were performed from 293.15 to 313.15 K for the neat DES and for caffeine solutions with molalities up to 0.701 mol kg^−1^. Instrument calibration was verified before each measurement series and after each sample by measuring distilled water over the same temperature interval. The standard uncertainties used in reporting the data were *u*(*d*) = 8.2 × 10^−5^ g∙cm^−3^, *u*(*u*) = 0.5 m∙s^−1^, and *u*(*T*) = 0.01 K.

#### 3.3.4. Viscosity Measurements and Verification of Newtonian Behavior

Dynamic viscosities were measured using a Brookfield DV II+ Pro rotational viscometer from AMETEK Brookfield (Massachusetts, MA, United States). Temperature was controlled with a Lauda E 100 thermostatic bath with a stability of ±0.01 K. Approximately 8 cm^3^ of the neat DES or caffeine-containing DES was transferred to the measurement chamber, and an SC4-18 spindle was immersed in the sample. The rotational speed was selected to keep the torque within the recommended operating range. The measuring cell was covered with a protective cap to minimize moisture uptake without interfering with spindle rotation. The instrument was calibrated with viscosity standards supplied by the manufacturer. Reported values are averages of consecutive readings recorded after signal stabilization. Measurements were performed from 293.15 to 313.15 K for caffeine molalities up to 0.701 mol kg^−1^. The manufacturer-specified accuracy of the viscometer was ±1% of the full-scale range in use.

Newtonian behavior was evaluated using the same instrument at *T* = 298.15 K and *p* = 1 × 10^5^ Pa for the neat DES and for the solution containing *m* = 0.597 mol∙kg^−1^ caffeine. The complete shear-rate protocol and the corresponding experimental data are provided in Table S14 and Figure S3 of the Supporting Information.

#### 3.3.5. Derived Properties and Data Treatment

The density data at fixed composition were represented by a linear temperature correlation, whereas their composition dependence at fixed temperature was described by a second-order polynomial:

(1) $$d=b_{o}+b_{1}T$$

(2) $$d=c_{o}+c_{1}m+c_{2}m^{2}.$$

The isobaric thermal expansion coefficient was obtained from the temperature dependence of density:

(3) $$\alpha_{p}=-\frac{1}{d}{\left(\frac{\partial d}{\partial T}\right)}_{p}=-{\left(\frac{\partial \ln d}{\partial T}\right)}_{p}$$

For the neat DES and caffeine-containing mixtures, the mean molar volume was calculated using:

(4) $$V_{m}=\frac{1000+mM_{2}}{d(\frac{1000}{{\bar{M}}_{DES}}+m)},$$

where $\bar{M}$_DES_ = 140.883 g∙mol^−1^ is the molar mass of the 2:1 menthol–resorcinol DES, *M*_2_ is the molar mass of caffeine, *m* is caffeine molality, and *d* is the experimental density of the solution. 

The apparent molar volume of caffeine, *V*_ϕ_, was calculated from the solution density *d*, the density of the pure DES *d*_1_, caffeine molality *m*, and caffeine molar mass *M*_2_ using:

(5) $$V_{\phi }=\frac{1000(d_{1}-d)}{mdd_{1}}+\frac{M_{2}}{d}.$$

The partial molar volumes of the DES, $\bar{V}$_1_, and caffeine, $\bar{V}$_2_, were calculated from the composition dependence of *V*_ϕ_:

(6) $${\bar{V}}_{1}=\frac{M_{1}}{d_{1}}-\frac{M_{1}m^{2}}{1000}{\left(\frac{\partial V_{\phi }}{\partial m}\right)}_{T,p,n_{2}},$$

(7) $${\bar{V}}_{2}=m{\left(\frac{\partial V_{\phi }}{\partial m}\right)}_{T,p,n_{1}}+V_{\phi }.$$

Apparent molar volumes were correlated using the Masson relation for nonelectrolyte solutions [45]:

(8) $$V_{\phi }=V_{\phi }^{o}+S_{V}m,$$

where *V*_ϕ_^o^ is the limiting apparent molar volume and *S*_v_ is the concentration coefficient. The temperature dependence of *V*_ϕ_^o^ was fitted by a second-order polynomial, and the limiting apparent molar expansibility was obtained by differentiation:

(9) $$V_{\phi }^{o}=a_{o}+a_{1}T+a_{2}T^{2},$$

(10) $$E_{\phi }^{o}={\left(\frac{\partial V_{\phi }^{o}}{\partial T}\right)}_{p}=a_{1}+2a_{2}T.$$

The isentropic compressibility of each sample was calculated from the Newton–Laplace relation:

(11) $$\kappa_{s}=\frac{1}{u^{2}d}.$$

For the neat DES, and caffeine-containing systems, the acoustically derived intermolecular free length was estimated using the commonly adopted empirical Jacobson relation [59]:

*L*_f_ = *K*_T_*κ*_s_^1/2^, (12)

*K*_T_ = (93.875 + 0.375*T*) × 10^−8^, (13)

where *T* is expressed in kelvin and the numerical value of *K*_T_ is tied to the unit convention of the empirical relation. *L*_f_ is therefore interpreted as an acoustically derived indicator of the intermolecular free space, including the transient cavities arising from the dynamic packing of the DES components, rather than as a direct geometrical measure of fixed molecular voids [59].

Using SI units, the apparent molar isentropic compressibility of caffeine was calculated as:

(14) $$\kappa_{\phi }=\left[\frac{\kappa_{s}d_{1}-{{\kappa_{s}}_{,}}_{1}d}{mdd_{1}}\right]+\kappa_{s}\frac{M_{2}}{d},$$

where *κ*_s,1_ is the isentropic compressibility of the pure DES, and *M*_2_ is the molar mass of caffeine. Limiting values were obtained by linear extrapolation to zero molality:

(15) $$\kappa_{\phi }=\kappa_{\phi }^{o}+S_{k}m.$$

The temperature dependence of viscosity at each caffeine molality was described by the Arrhenius equation:

(16) $$\eta =\eta_{A}exp\left(\frac{E_{\eta }}{RT}\right),$$

which can be written in linear form as:

$$\ln\eta =\ln\eta_{A}+\frac{E_{\eta }}{RT},$$

where *η*_A_ is the pre-exponential factor, *E*_η_ is the apparent activation energy of viscous flow, *R* is the universal gas constant, and *T* is the absolute temperature. The *E*_η_ values were obtained from the slopes of the linear dependences of ln*η* on 1/*T*.

For descriptive comparison of the concentration dependence of viscosity, caffeine molality was first converted to molar concentration, *c*, using the measured density and the caffeine molar mass:

*c* = 1000*md*/(1000 + *mM*_2_). (17)

The effect of caffeine addition on the viscous behavior of the DES was quantified using the viscosity *B*-coefficient obtained from the simplified Jones–Dole relation for nonelectrolyte solutions [52]:

(18) $$\frac{\eta }{\eta_{o}}-1=Bc,$$

where *η* and *η*_o_ denote the dynamic viscosities of the caffeine-containing system and the pure DES, respectively, while *c* is the caffeine molar concentration (mol∙dm^−3^).

The activation parameters for viscous flow were further evaluated using the Eyring–Feakins approach [58,60]. The standard molar Gibbs free energy of activation for viscous flow of the neat DES, $\Delta \mu_{1}^{o“\neq ”‡}$, was calculated from:

(19) $$\Delta {\mu_{1}}^{o\neq }=RT\ln\left(\frac{\eta_{o}{\bar{V}}_{1}^{o}}{hN_{A}}\right),$$

where ${\bar{V}}_{1}^{o}$ is the molar volume of the neat DES, h is Planck’s constant, and $N_{A}$ is Avogadro’s constant.

The standard molar Gibbs free energy of activation associated with caffeine at infinite dilution, $\Delta \mu_{2}^{o“\neq ”}$, was calculated using the Feakins relation:

(20) $$\Delta \mu_{2}^{o\neq }=\Delta \mu_{1}^{o\neq }+\frac{RT}{{\bar{V}}_{1}^{o}}\left(B+{\bar{V}}_{2}^{o}-{\bar{V}}_{1}^{o}\right),$$

where ${\bar{V}}_{2}^{o}$ is the limiting partial molar volume of caffeine, equal to $V_{\phi }^{o}$. In Equation (20), B, ${\bar{V}}_{1}^{o}$, and ${\bar{V}}_{2}^{o}$ were expressed in consistent volumetric units.

The standard molar entropy of activation associated with caffeine, $\Delta S_{2}^{o“\neq ”}$, was obtained from the temperature dependence of $\Delta \mu_{2}^{o“\neq ”}$:

(21) $$\Delta {S_{2}}^{o\neq }=-\frac{\partial \left(\Delta {\mu_{2}}^{o\neq }\right)}{\partial T}.$$

The corresponding standard molar enthalpy of activation, $\Delta H_{2}^{o“\neq ”}$, was calculated from:

(22) $$\Delta {H_{2}}^{o\neq }=\Delta {\mu_{2}}^{o\neq }+T\Delta S_{2}^{o\neq }.$$

#### 3.3.6. Computational Procedure

Density functional theory calculations were performed using the Jaguar 8.8 program within the Schrödinger Materials Science Suite. Two representative finite-cluster models were considered. The first cluster consisted of two menthol molecules and one resorcinol molecule, corresponding to the 2:1 molar composition of the investigated DES. The second cluster contained the same DES components together with one caffeine molecule. The geometries of isolated caffeine and both molecular clusters were optimized at the B3LYP/6-311G(d,p) level of theory [61,62,63]. Molecular electrostatic potential surfaces were generated at the same level to identify electron-rich, electron-deficient, and less-polar molecular regions. Selected intermolecular distances were extracted from the optimized structures.

## 4. Conclusions

The thermal results identify the 2:1 menthol-to-resorcinol ratio as the experimentally observed eutectic minimum within the investigated composition series. The large melting-point depression enables two initially crystalline compounds to form a liquid phase under ambient conditions. The DFT-optimized DES cluster provides a plausible molecular representation of this behavior, in which one resorcinol molecule connects two menthol molecules through simultaneous heteromolecular hydrogen bonds.

The combined volumetric, acoustic, and viscosity results demonstrate that caffeine is not simply dissolved in the available free volume of the DES but induces a concentration- and temperature-dependent reorganization of its local environment. At low caffeine content, the shallow minima in molar volume and viscosity indicate efficient accommodation of the first added caffeine molecules. The decrease in the limiting apparent molar volume with temperature, which was not observed for caffeine in water, ethylene glycol, or methyl salicylate, further distinguishes the menthol–resorcinol DES as a specific solvation environment. Caffeine also reduces the thermal expansivity, isentropic compressibility, and acoustically derived intermolecular free length of the liquid. At higher molalities, concentration-dependent contributions become increasingly important, suggesting a growing influence of caffeine–caffeine contacts and collective rearrangement of the DES components.

The pronounced increase in viscosity at higher caffeine contents, together with the exceptionally large positive Jones–Dole *B*-coefficients and activation parameters of viscous flow, shows that caffeine strongly restricts the molecular rearrangements required for flow. This behavior may be described as an apparent structure-making response, although the limitations of transferring this terminology from aqueous solutions to a nonaqueous multicomponent liquid must be recognized. The DFT results provide a complementary local interpretation. Caffeine is incorporated into the interaction network without disrupting the principal menthol–resorcinol hydrogen bonds, forming an additional O–H···O=C hydrogen bond with menthol. The MEP surfaces further reveal electrostatic complementarity between the polar and less-polar regions of caffeine and the chemically heterogeneous DES environment.

A particularly important practical result is that clear homogeneous solutions containing 136 g of caffeine per kilogram of DES can be prepared. The combination of hydrophilic resorcinol and hydrophobic menthol therefore creates a dual interaction environment capable of accommodating the polar and less-polar regions of an amphiphilic solute. Such chemically heterogeneous liquids may be useful not only for caffeine but also for other amphiphilic pharmaceuticals, alkaloids, and natural bioactive compounds that are insufficiently soluble in exclusively polar or nonpolar solvents. Potential applications include selective extraction from complex matrices, preparation of highly concentrated liquid formulations, and topical or transdermal delivery. In topical systems, menthol may further facilitate skin penetration, while the relatively high viscosity of the DES may increase residence time at the site of application. However, its effect on diffusion and release must also be considered. Direct measurements of equilibrium solubility, extraction selectivity, release kinetics, skin permeation, and biocompatibility are therefore required before a specific pharmaceutical or cosmetic application can be proposed. In practical topical applications, this DES could serve as a concentrated caffeine-solubilizing base, subsequently combined with cosmetically acceptable diluents and excipients to achieve final concentrations of caffeine, menthol, and resorcinol appropriate for the intended formulation and its safety and performance requirements.

Conventional solvents may remain more economical when evaluated solely on the basis of their bulk price. However, the high caffeine-loading capacity and simple preparation of the menthol–resorcinol DES may reduce the required solvent volume and facilitate the preparation of concentrated formulations. Its overall cost-effectiveness should therefore be evaluated for a specific process or product rather than based solely on solvent price. Overall, the menthol–resorcinol DES represents a promising and tunable platform for the solubilization and delivery of amphiphilic compounds rather than merely an alternative solvent for caffeine.

## Figures and Tables

**Table 1:** Parameters obtained from fitting the apparent molar volumes of caffeine + DES solutions with the Masson equation for nonelectrolytes (Equation (8)) in the temperature range 293.15–313.15 K, together with the standard deviation of the fit (*σ*) and the regression coefficient (*R*^2^).

*T*/(K)	$V_{\phi }^{o}$/(cm^3^·mol^−1^)	*S*_v_/(cm^3^·kg·mol^−2^)	*σ*	*R* ^2^
293.15	141.04	2.60	0.03	0.9976
298.15	139.86	4.74	0.11	0.9903
303.15	137.09	9.44	0.26	0.9868
308.15	135.29	12.3	0.27	0.9911
313.15	132.70	16.5	0.16	0.9984

**Table 2:** Acoustically derived intermolecular free length, *L*_f_, for the pure menthol–resorcinol DES and caffeine solutions at different temperatures.

*m*/(mol·kg^−1^)	*L*_f_·10^11^/(m)				
*T*/K	293.15	298.15	303.15	308.15	313.15
0.000	4.210	4.404	4.551	4.675	4.789
0.100	4.192	4.386	4.535	4.660	4.774
0.199	4.162	4.359	4.511	4.638	4.751
0.300	4.136	4.335	4.491	4.619	4.731
0.401	4.111	4.313	4.471	4.600	4.712
0.501	4.087	4.290	4.451	4.581	4.692
0.584	4.068	4.271	4.434	4.564	4.676
0.701	4.041	4.245	4.411	4.542	4.653

**Table 3:** Parameters obtained from fitting data for caffeine in DES using Equation (15) at different temperatures, including the intercept *κ*_ϕ_, slope *S*_k_, standard deviation σ, and regression coefficient *R*^2^.

*T*/(K)	$\kappa_{\phi }^{o}$·10^15^/(m^3^·mol^−1^∙Pa^−1^)	*S*_k_·10^15^/(m^3^·kg∙mol^−2^·Pa^−1^)	*σ*	*R* ^2^
293.15	4.049	1.70	0.06	0.9758
298.15	14.69	−1.54	0.07	0.9675
303.15	25.82	−3.73	0.08	0.9917
308.15	30.84	−3.82	0.09	0.9904
313.15	32.21	−4.31	0.12	0.9851

**Table 4:** Jones–Dole viscosity *B*-coefficients for caffeine in the menthol–resorcinol DES, ethylene glycol, water, and methyl salicylate in the temperature range *T* = 293.15–313.15 K. Literature values are from refs. [46,47,48].

*T/*(K)	*B*/(dm^3^·mol^−1^)			
	DES	Ethylene Glycol	Water	Methyl Salicylate
293.15	2.122	−0.2640	0.414	0.684
298.15	1.742	−0.2789	0.421	0.626
303.15	1.587	−0.3049	0.429	0.588
308.15	1.419	−0.3038	0.432	0.555
313.15	1.308	−0.3213	0.435	0.514

**Table 5:** Calculated values of $\Delta S_{2}^{o\neq }$ and $\Delta H_{2}^{o\neq }$ for caffeine in different systems. Literature values are from refs. [46,47,48].

System	$\Delta S_{2}^{o\neq }$/(J∙mol^−1^∙K^−1^)	$\Delta H_{2}^{o\neq }$/(kJ∙mol^−1^)
Caffeine + DES	875.1	325.3
Caffeine + ethylene glycol	222.9	77.46
Caffeine + water	−407.7	37.67
Caffeine + methyl salicylate	132.5	68.88

**Table 6:** Compounds used in the study with main specifications.

Compound	Chemical Structure	Abbreviation	Supplier	CAS Number	Mass Fraction Purity (wt%)
(−)-Menthol	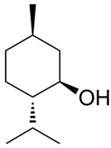	Men	Sigma-Aldrich	2216–51–5	≥99
Resorcinol	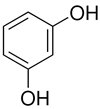	Res	Sigma-Aldrich	108-46–3	≥99
Caffeine	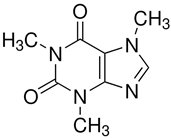	Caf	Sigma-Aldrich	58–08–2	≥99

## Data Availability

The original contributions presented in this study are included in the article and Supplementary Materials. Further inquiries can be directed to the corresponding author.

## Supplementary Materials

The following supporting information can be downloaded at: https://www.mdpi.com/article/10.3390/molecules31173039/s1, Table S1: Endset temperatures of the final melting transition and mass-normalized enthalpies of the low-temperature eutectic melting event in the menthol–resorcinol system; Table S2: Onset temperatures of mass loss, Tonset, for the pure components, the neat menthol–resorcinol DES, and caffeine-containing systems; Table S3: Molality, (m) and density, (d) of caffeine + DES solutions in the temperature range from T = (293.15 to 313.15) K at p = 1·10^5^ Pa; Table S4: Fitting parameters of the plot representing density as a function of the temperature (Equation (1)) for each caf-feine molality (m), the standard deviation (σ) of the fit with the regression coefficient (R2); Table S5: Fitting parameters of the plot representing density as a function of the caffeine molality (m) (Equation (2)), for each temperature, the standard deviation (σ) of the fit with the regression coefficient (R2); Table S6: Values of thermal expansion coefficient, (α_p_), for caffeine + DES solutions for each caffeine molality (m) in the temperature range from T = (293.15 to 313.15) K at p = 1·10^5^ Pa; Table S7: Molar volume of caffeine + DES solutions in the temperature range from T = (293.15 to 313.15) K at p = 1·10^5^ Pa. Table S8. Apparent molar volume, (V_ϕ_), partial molar volume of DES ($\bar{V}$_1_) and partial molar volume of caffeine, ($\bar{V}$_2_), in caffeine + DES mixture in the temperature range from T = (293.15 to 313.15) K at p = 1·10^5^ Pa; Table S9: Fitting coefficients ao, a1 and a2 of apparent molar volumes, (V_ϕ_), of the caffeine + DES mixtures (Equation (9)), with the regression coefficient (R^2^); Table S10: Values of the limiting apparent molar expansibilities, ($E_{\phi }^{o}$) of the caffeine + DES solutions (Equation (10)) in the temperature range from T = (293.15 to 313.15) K; Table S11: The speed of sound and calculated values of isentropic compressibility, (κs) of caffeine in DES solution in the temperature range from T = (293.15 to 313.15) K; Table S12: Calculated values of apparent molar isentropic compressibility, (κϕ), of caffeine in DES solution in the temperature range from T = (293.15 – 313.15) K at p = 1 · 10^5^ Pa; Table S13: Dynamic viscosity of caffeine + DES solutions in the temperature range from T = (293.15 to 313.15) K at p = 1·10^5^ Pa; Table S14: Values of viscosity, shear stress, shear rate and revolution per minute of spindle (RPM), at two caffeine molalities and at T = 298.15 K and p = 1·10^5^ Pa; Table S15: Arrhenius parameters for viscous flow of the pure menthol–resorcinol DES and caffeine-containing sys-tems at different caffeine molalities, calculated using Equation (16): logarithm of the pre-exponential factor, lnηA, activa-tion energy of viscous flow (*E**η*), standard deviation (σ) and regression coefficient (*R*^2^); Table S16: Thermodynamic parameters of viscous flow in the temperature range T = (293.15 to 313.15) K; Table S17: Selected intermolecular contacts and corresponding distances in the DFT-optimized menthol–resorcinol and menthol–resorcinol–caffeine clusters; Figure S1: DSC heating curves of menthol–resorcinol binary mixtures. The curves are vertically shifted for clarity; Figure S2: Dependence of density, *d*, of the neat menthol–resorcinol DES and caffeine-containing systems on (a) temperature at different caffeine molalities, (m): = (■) 0.0000, (●) 0.100, (▲) 0.199, (▼) 0.300, (◆) 0.401, (◀) 0.501, (▶) 0.584 and (⬢) 0.701; (b) caffeine molality, at different temperatures, (T): = (■) 293.15; (●) 298.15; (▲) 303.15; (▼) 308.15 and (◆) 313.15. Symbols represent the experimental data, while the lines represent fits obtained using Equations (1) and (2); Figure S3: Verification of Newtonian behavior for pure DES and caffeine solution at m = 0.597 mol∙kg^−1^ at T = 298.15 K: (a) dynamic viscosity as a function of shear rate and (b) shear stress as a function of shear rate; Figure S4: Dependence of lnη on reciprocal absolute temperature (1/*T*) for the neat menthol–resorcinol DES and caffeine-containing systems at different caffeine molalities (m): = (■) 0.0000, (●) 0.100, (▲) 0.199, (▼) 0.300, (◆) 0.401, (◀) 0.501, (▶) 0.597 and (⬢) 0.701. The solid lines represent fits according to the Arrhenius equation (Equation (16)); Figure S5: Plot of specific viscosity, ($\frac{\eta }{\eta_{o}}-1$ ), against concentration, (c), of the caffeine + DES mixture, at different temper-atures: T = (■) 293.15; (●) 298.15; (▲) 303.15; (▼) 308.15 and (◆) 313.15. The points represent experimental values and lines represent values calculated from Equation (18); Figure S6: Molecular electrostatic potential surface of caffeine calculated at the B3LYP/6-311G(d,p) level of theory. Red and orange regions represent negative electrostatic potential, green regions indicate values close to zero, and blue and purple regions represent positive electrostatic potential; Figure S7: Molecular electrostatic potential surface of the representative DES cluster composed of two menthol molecules and one resorcinol molecule, calculated at the B3LYP/6-311G(d,p) level of theory. Red and orange regions represent negative electrostatic potential, green regions indicate values close to zero, and blue and purple regions represent positive electrostatic potential; Figure S8: Molecular electrostatic potential surface of the representative cluster composed of two menthol mole-cules, one resorcinol molecule, and one caffeine molecule, calculated at the B3LYP/6-311G(d,p) level of theory. Red and orange regions represent negative electrostatic potential, green regions indicate values close to zero, and blue and purple regions represent positive electrostatic potential.
