# Supplementary material for: Thermophysical Behavior and Molecular Interactions of Caffeine in a Novel Menthol–Resorcinol Type V Deep Eutectic Solvent
Source: Molecules. 2026 Aug 29;31(17):3039. doi: 10.3390/molecules31173039 (PMC13567191; doi:10.3390/molecules31173039)
Supplement: Supplementary file 1 [file molecules-31-03039-s001.zip › molecules-4504695-supplementary.pdf]

Thermophysical Behavior and Molecular Interactions of Caffeine in a Novel Menthol–Resorcinol Type V Deep Eutectic Solvent

Milan Vraneš[1]*, Snežana Papović[1], Janez Cerar[2], Romana Cerc Korošec[2], Teona Teodora Borović[1], Jasmin Suljagić[3], Edita Bjelić[3], Mersiha Suljkanović[4] and David Nedeljković[1]

[1]*Faculty of Sciences, Department of Chemistry, Biochemistry and Environmental Protection,*

University of Novi Sad, Trg Dositeja Obradovića 3, 21000 Novi Sad, Serbia

[2]*Faculty of Chemistry and Chemical Technology, University of Ljubljana, Večna pot 113, 1000 Ljubljana, Slovenia*

[3]*Faculty of Technology, University of Tuzla, Urfeta Vejzagića 8, 75000 Tuzla, Bosnia and Herzegovina*

[4]*Faculty of Natural Sciences and Mathematics, University of Tuzla, Urfeta Vejzagića 4, 75000 Tuzla, Bosnia and Herzegovina*

Corresponding author: Milan Vraneš (milan.vranes@dh.uns.ac.rs)

Table S1. Endset temperatures of the final melting transition and mass-normalized enthalpies of the low-temperature eutectic melting event in the menthol–resorcinol system.

x(Men)	T_{endset} / (°C)	ΔH_{eut} / (J·g^{-1})
0.0000	108.5	0.00[a]
0.1407	105.3	0.23
0.2705	104.3	0.47
0.3788	97.0	1.02
0.4817	85.1	1.47
0.5841	30.2	2.13
0.6001	-25.1	2.46
0.6667	-36.5	2.84
0.7660	-27.2	1.69
0.8828	12.1	1.21
0.9248	34.3	0.71
1.0000	41.4	0.00[a]

[a]For pure resorcinol (x(Men) = 0) and pure (−)-menthol (x(Men) = 1), ΔH_{eut} was set to zero because the eutectic melting event is absent. These values do not represent the total enthalpies of fusion of the pure components.

Table S2. Onset temperatures of mass loss, T_{onset}, for the pure components, the neat menthol–resorcinol DES, and caffeine-containing systems.

Name and abbreviation	T_{onset} / (°C)
Menthol (Men)	111
Resorcinol (Res)	145
Men:Res 2:1	123
0.100 mol·kg^{-1} Caf in Men:Res 2:1	100
0.401 mol·kg^{-1} Caf in Men:Res 2:1	107
0.701 mol·kg^{-1} Caf in Men:Res 2:1	121
Caffeine (Caf)	224

Table S3. Molality, (m) and density, (d) of caffeine + DES solutions in the temperature range from T = (293.15 to 313.15) K at $p = 1\cdot10^5$ Pa.

m / (mol·kg^{-1})	d / (g·cm^{-3})				
T / (K)	293.15	298.15	303.15	308.15	313.15
0.000	0.978791	0.975063	0.971201	0.967336	0.963418
0.100	0.984190	0.980595	0.977001	0.973301	0.969591
0.199	0.989332	0.985799	0.982315	0.978716	0.975214
0.300	0.994415	0.990899	0.987475	0.983999	0.980547
0.401	0.999260	0.995770	0.992329	0.988798	0.985356
0.501	1.003916	1.000378	0.996831	0.993298	0.989733
0.584	1.007705	1.004105	1.000455	0.997001	0.993078
0.701	1.012760	1.009101	1.005210	1.001303	0.997528

Standard uncertainties are: $u(d) = 8.2\cdot10^{-5}$ g·cm^{-3}, $u(m) = 3.5\cdot10^{-4}$ mol·kg^{-1}, $u(T)$ = 0.01 K;
Relative standard uncertainty: $u_r(p)$=0.015.

Table S4. Fitting parameters of the plot representing density as a function of the temperature (eq. 1) for each caffeine molality (m), the standard deviation (σ) of the fit with the regression coefficient (R^2).

m / (mol·kg^{-1})	b_o / (g·cm^{-3})	b_1·10^4 / (g·cm^{-3}·K^{-1})	σ·10^5	R^2
0.000	1.20442	-7.69	6.219	0.9999
0.100	1.19819	-7.30	5.374	0.9999
0.199	1.19641	-7.06	2.858	0.9999
0.300	1.19747	-6.93	2.285	0.9999
0.401	1.20317	-6.96	2.315	0.9999
0.501	1.21174	-7.09	0.885	0.9999
0.584	1.22091	-7.27	12.09	0.9997
0.701	1.23716	-7.65	7.683	0.9998

The coefficients obtained from equation (1).

Table S5. Fitting parameters of the plot representing density as a function of the caffeine molality (m) (eq. 2), for each temperature, the standard deviation (σ) of the fit with the regression coefficient (R^2).

T / (K)	c_o / (g·cm^{-3})	c_1·10^2 / (g·kg·cm^{-3}·mol^{-1})	c_2·10^3 / (g·kg^2·cm^{-3}·mol^{-2})	σ·10^5	R^2
293.15	0.97880	5.461	-8.810	1.19	0.9999
298.15	0.97509	5.576	-10.38	2.86	0.9999
303.15	0.97126	5.821	-14.02	5.07	0.9999
308.15	0.96736	6.052	-17.16	7.89	0.9997
313.15	0.96348	6.283	-20.51	7.93	0.9996

The coefficients obtained from equation (2).

Table S6. Values of thermal expansion coefficient, (α_p), for caffeine + DES solutions for each caffeine molality (m) in the temperature range from T = (293.15 to 313.15) K at $p = 1\cdot10^5$ Pa.

$\alpha_p\cdot10^4$ / (K^{-1})								
m / (mol·kg^{-1})	0.000	0.100	0.199	0.300	0.401	0.501	0.584	0.701
	7.924	7.471	7.191	7.015	7.010	7.112	7.269	7.424

Table S7. Molar volume of caffeine + DES solutions in the temperature range from T = (293.15 to 313.15) K at $p = 1\cdot10^5$ Pa.

m / (mol·kg^{-1})	V_m / (cm^3·mol^{-1})				
T / (K)	293.15	298.15	303.15	308.15	313.15
0.000	143.93	144.48	145.06	145.64	146.23
0.100	143.90	144.42	144.95	145.51	146.06
0.199	143.87	144.38	144.90	145.43	145.95
0.300	143.85	144.36	144.86	145.37	145.88
0.401	143.83	144.34	144.84	145.36	145.86
0.501	143.83	144.34	144.85	145.37	145.89
0.584	143.83	144.34	144.87	145.37	145.95
0.701	143.84	144.36	144.92	145.48	146.03

Table S8. Apparent molar volume, (V_ϕ), partial molar volume of DES ($\overline{V}_1$) and partial molar volume of caffeine, ($\overline{V}_2$), in caffeine + DES mixture in the temperature range from T = (293.15 to 313.15) K at $p = 1\cdot10^5$ Pa.

m / (mol·kg^{-1})	V_ϕ / (cm^3·mol^{-1})				
T / (K)	293.15	298.15	303.15	308.15	313.15
0.100	141.26	140.17	137.63	136.15	134.19
0.199	141.59	140.87	139.16	138.02	136.05
0.300	141.85	141.42	140.17	139.08	137.69
0.401	142.09	141.78	140.96	140.38	139.39
0.501	142.35	142.27	141.92	141.52	141.07
0.584	142.54	142.63	142.58	142.14	142.49
0.701	142.86	143.09	143.49	143.91	144.04
m / (mol·kg^{-1})	$\overline{V}_1$ / (cm^3·mol^{-1})				
0.100	143.93	144.48	145.04	145.62	146.21
0.199	143.92	144.46	145.00	145.57	146.14
0.300	143.90	144.42	144.94	145.48	146.02
0.401	143.87	144.38	144.84	145.36	145.86
0.501	143.84	144.32	144.72	145.20	145.65
0.584	143.81	144.26	144.60	145.05	145.44
0.701	143.75	144.16	144.40	144.79	145.09
m / (mol·kg^{-1})	$\overline{V}_2$ / (cm^3·mol^{-1})				
0.100	141.52	140.82	139.27	138.15	136.00
0.199	142.11	141.81	141.03	140.46	139.33
0.300	142.63	142.84	143.00	142.76	142.65
0.401	143.13	143.67	144.75	145.29	146.00
0.501	143.65	144.64	146.64	147.66	149.34
0.584	144.06	145.40	148.09	149.30	152.14
0.701	144.68	146.41	150.10	152.50	155.61

Standard uncertainties are: $u(d) = 8.2\cdot10^{-5}$ g·cm^{-3}, $u(m) = 3.5\cdot10^{-4}$ mol·kg^{-1}, $u(T)$ = 0.01 K, $u(V_\phi) = 5.6\cdot10^{-3}$ cm^3·mol^{-1}; $u(\overline{V}_2) = 6.9\cdot10^{-3}$ cm^3·mol^{-1};

Relative standard uncertainty: $u_r(p)$=0.015.

Table S9. Fitting coefficients a_o, a_1 and a_2 of apparent molar volumes, (V_ϕ), of the caffeine + DES mixtures (eq. 9), with the regression coefficient (R^2).

a_o / (cm^3·mol^{-1})	a_1 / (cm^3·mol^{-1}·K^{-1})	a_2 / (cm^3·mol^{-1}·K^{-2})	R^2
-219.45	2.780	-0.0053	0.9927

Table S10. Values of the limiting apparent molar expansibilities, (E_ϕ^o) of the caffeine + DES solutions (eq. 10) in the temperature range from T = (293.15 to 313.15) K.

	E_ϕ^o / (cm^3·mol^{-1}·K^{-1})				
T / (K)	293.15	298.15	303.15	308.15	313.15
	-0.3042	-0.3568	-0.4094	-0.4620	-0.5146

Table S11. The speed of sound and calculated values of isentropic compressibility, (κ_s) of caffeine in DES solution in the temperature range from T = (293.15 to 313.15) K.

m / mol·kg^{-1}	T / K					T / K				
	293.15	298.15	303.15	308.15	313.15	293.15	298.15	303.15	308.15	313.15
	u / m·s^{-1}					$\kappa_s \cdot 10^{12}$ / Pa^{-1}				
0.000	1545.67	1495.36	1463.88	1441.13	1422.60	427.64	458.64	480.49	497.76	512.88
0.100	1551.29	1498.97	1465.48	1441.87	1422.98	422.22	453.86	476.59	494.20	509.35
0.199	1556.92	1502.81	1467.64	1443.25	1423.95	416.99	449.16	472.62	490.52	505.72
0.300	1562.72	1506.99	1470.15	1444.98	1425.48	411.78	444.38	468.54	486.72	501.89
0.401	1568.33	1511.13	1472.96	1447.32	1427.73	406.86	439.78	464.48	482.80	497.87
0.501	1574.15	1515.45	1476.27	1449.87	1430.49	401.99	435.26	460.31	478.92	493.76
0.584	1578.83	1519.32	1479.05	1452.05	1433.17	398.10	431.44	456.92	475.71	490.25
0.701	1585.65	1524.76	1483.26	1456.21	1437.12	392.72	426.25	452.18	470.96	485.39

Table S12. Calculated values of apparent molar isentropic compressibility, (κ_ϕ), of caffeine in DES solution in the temperature range from T = (293.15 – 313.15) K at p = 1 x 10^5 Pa.

m / mol·kg^{-1}	$\kappa_\phi \cdot 10^{15}$ / m^3·mol^{-1}·Pa^{-1}				
T / K	293.15	298.15	303.15	308.15	313.15
0.100	4.24	14.57	25.48	30.49	31.62
0.199	4.37	14.41	25.06	30.13	31.43
0.300	4.49	14.13	24.75	29.72	31.12
0.401	4.82	14.06	24.32	29.16	30.49
0.501	4.86	14.02	23.81	28.87	29.99
0.584	5.10	13.79	23.61	28.61	29.66
0.701	5.20	13.59	23.30	28.26	29.20

Table S13. Dynamic viscosity of caffeine + DES solutions in the temperature range from T = (293.15 to 313.15) K at $p = 1\cdot10^5$ Pa.

m / (mol·kg^{-1})	η / (mPa·s)				
T / (K)	293.15	298.15	303.15	308.15	313.15
0.000	3808.6	1746.9	829.39	386.32	214.15
0.100	3766.5	1552.2	751.48	383.82	216.69
0.199	4407.2	1819.6	876.06	438.30	241.82
0.300	5121.4	2087.5	1004.0	478.47	265.55
0.401	5818.9	2356.6	1090.3	531.32	288.98
0.501	6566.1	2622.4	1225.8	576.24	315.43
0.597	7247.8	2881.9	1336.9	623.00	340.68
0.701	8013.3	3158.6	1448.3	676.99	363.52

Viscometer accuracy: ±1% of the full-scale range in use.
Standard uncertainties: $u(T)$ = 0.01 K, $u(m) = 3.5\cdot10^{-4}$ mol·kg^{-1}
Relative standard uncertainty: $u_r(p)$ = 0.015.

Table S14. Values of viscosity, shear stress, shear rate and revolution per minute of spindle (RPM), at two caffeine molalities and at T = 298.15 K and $p = 1 \cdot 10^5$ Pa.

RPM	η / (mPa·s)	Shear stress / (N·m^{-2})	Shear rate / (s^{-1})
		m = 0.000 mol·kg^{-1}	
1.5	1749.4	3.21	1.98
1.4	1748.0	2.97	1.85
1.2	1750.7	2.55	1.58
1.1	1747.3	2.33	1.45
1	1741.4	2.11	1.32
0.9	1738.6	1.90	1.19
0.8	1741.6	1.69	1.06
0.7	1738.6	1.48	0.92
0.6	1736.2	1.26	0.79
0.5	1739.1	1.05	0.66
0.4	1732.9	0.84	0.53
0.3	1729.2	0.63	0.40
0.2	1725.4	0.41	0.26
		m = 0.597 mol·kg^{-1}	
0.9	2898.4	2.97	1.19
0.8	2923.4	2.68	1.06
0.7	2919.7	2.35	0.92
0.6	2885.6	1.97	0.79
0.5	2890.7	1.65	0.66
0.4	2886.5	1.31	0.53
0.3	2874.5	0.98	0.40
0.2	2881.4	0.64	0.26

Viscometer accuracy: ±1% of the full-scale range in use.
Standard uncertainties: $u(T)$ = 0.01 K;
Relative standard uncertainties: $u_r(p)$ = 0.015.

For the neat DES, the spindle speed was varied from 0.2 to 1.5 rpm, corresponding to shear rates from 0.26 to 1.98 s^{-1}. For the caffeine-containing solution, the speed was varied from 0.2 to 0.9 rpm, corresponding to shear rates from 0.26 to 1.19 s^{-1}. Dynamic viscosity and shear stress were recorded after stabilization at each speed.

Table S15. Arrhenius parameters for viscous flow of the pure menthol–resorcinol DES and caffeine-containing systems at different caffeine molalities, calculated using Eq. (16): logarithm of the pre-exponential factor, $\ln\eta_A$, activation energy of viscous flow (E_η), standard deviation (σ) and regression coefficient (R^2).

m / (mol·kg^{-1})	ln(η_A / (mPa·s))	E_η / (kJ·mol^{-1})	σ	R^2
0.000	-37.30	111.0	0.043	0.9990
0.100	-36.43	108.7	0.081	0.9961
0.199	-37.01	110.5	0.071	0.9971
0.300	-37.87	113.0	0.071	0.9972
0.401	-38.40	114.5	0.073	0.9972
0.501	-38.85	115.9	0.074	0.9972
0.597	-39.13	116.9	0.075	0.9971
0.701	-39.52	118.1	0.073	0.9973

Table S16. Thermodynamic parameters of viscous flow in the temperature range T = (293.15 to 313.15) K.

Parameters					
T / (K)	293.15	298.15	303.15	308.15	313.15
$\bar{V}_2^{o}$ / (cm^3·mol^{-1})	141.04	139.86	137.09	135.29	132.70
$\bar{V}_1^{o}$ / (cm^3·mol^{-1})	143.98	144.56	145.22	145.84	146.49
$\Delta\mu_1^{o\neq}$ / (kJ·mol^{-1})	34.45	33.11	31.80	30.38	29.35
$\Delta\mu_2^{o\neq}$ / (kJ·mol^{-1})	70.32	62.91	59.21	55.11	52.34

Table S17. Selected intermolecular contacts and corresponding distances in the DFT-optimized menthol–resorcinol and menthol–resorcinol–caffeine clusters.

Cluster	Molecular pair	Contact	Distance / Å	Geometrical interpretation
DES	Resorcinol–menthol 1	O30–H76···O19	1.72	O–H···O hydrogen bond
DES	Resorcinol–menthol 2	O29–H75···O8	1.72	O–H···O hydrogen bond
DES	Resorcinol–menthol 1	H73···O19	2.75	Possible weak C–H···O contact
DES	Menthol 1–resorcinol	H66···O30	2.85	Long, weak C–H···O contact
DES	Menthol 2–resorcinol	H46···O29	2.86	Long, weak C–H···O contact
DES	Resorcinol–menthol 2	H72···O8	2.66	Possible weak C–H···O contact
DES–caffeine	Resorcinol–menthol 1	O30–H76···O19	1.65	O–H···O hydrogen bond
DES–caffeine	Resorcinol–menthol 2	O29–H75···O8	1.76	O–H···O hydrogen bond
DES–caffeine	Menthol 1–caffeine	O19–H63···O10	1.72	O–H···O=C hydrogen bond
DES–caffeine	Caffeine–resorcinol	H24···O29	2.24	Possible weak C–H···O contact
DES–caffeine	Caffeine–menthol 1	H17···O19	2.78	Possible weak C–H···O contact
DES–caffeine	Resorcinol–caffeine	H73···O10	2.75	Possible weak C–H···O contact
DES–caffeine	Menthol 1–resorcinol	H66···O30	3.19	Long geometrical contact
DES–caffeine	Resorcinol–menthol 2	H72···O8	2.61	Possible weak C–H···O contact

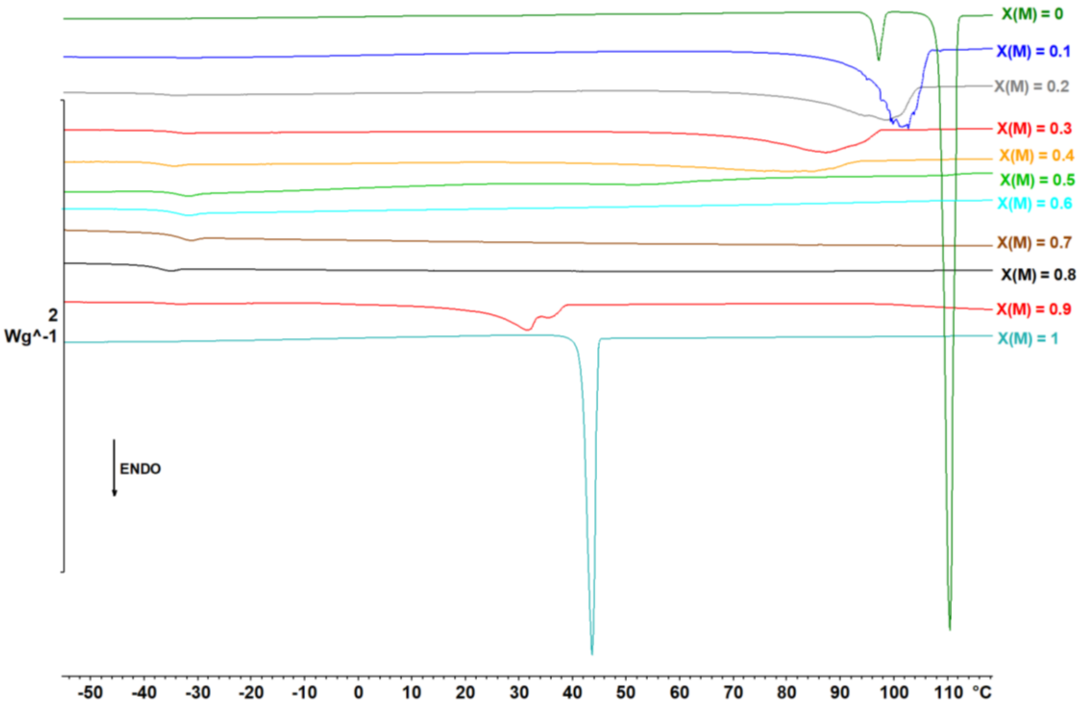

Figure S1. DSC heating curves of menthol–resorcinol binary mixtures. The curves are vertically shifted for clarity.

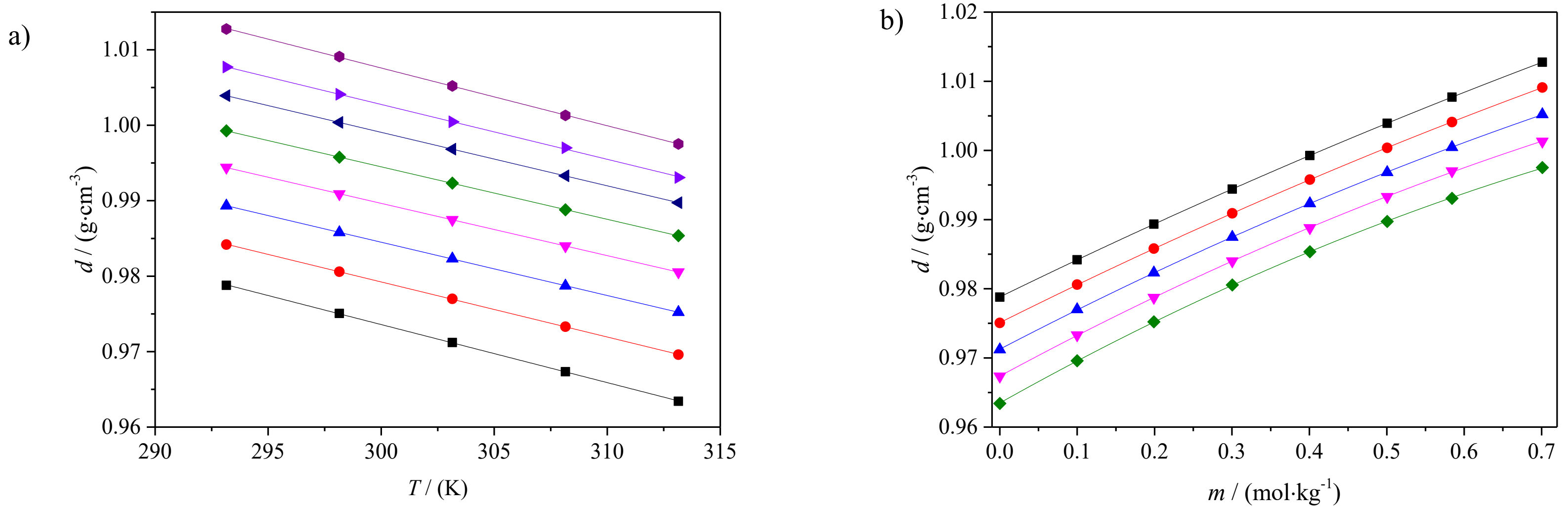

Figure S2. Dependence of density, d, of the neat menthol–resorcinol DES and caffeine-containing systems on (a) temperature at different caffeine molalities, (m): = (■) 0.0000, (●) 0.100, (▲) 0.199, (▼) 0.300, (◆) 0.401, (◀) 0.501, (▶) 0.584 and (⬢) 0.701; b) caffeine molality, at different temperatures, (T): = (■) 293.15; (●) 298.15; (▲) 303.15; (▼) 308.15 and (◆) 313.15. Symbols represent the experimental data, while the lines represent fits obtained using Eqs. (1) and (2).

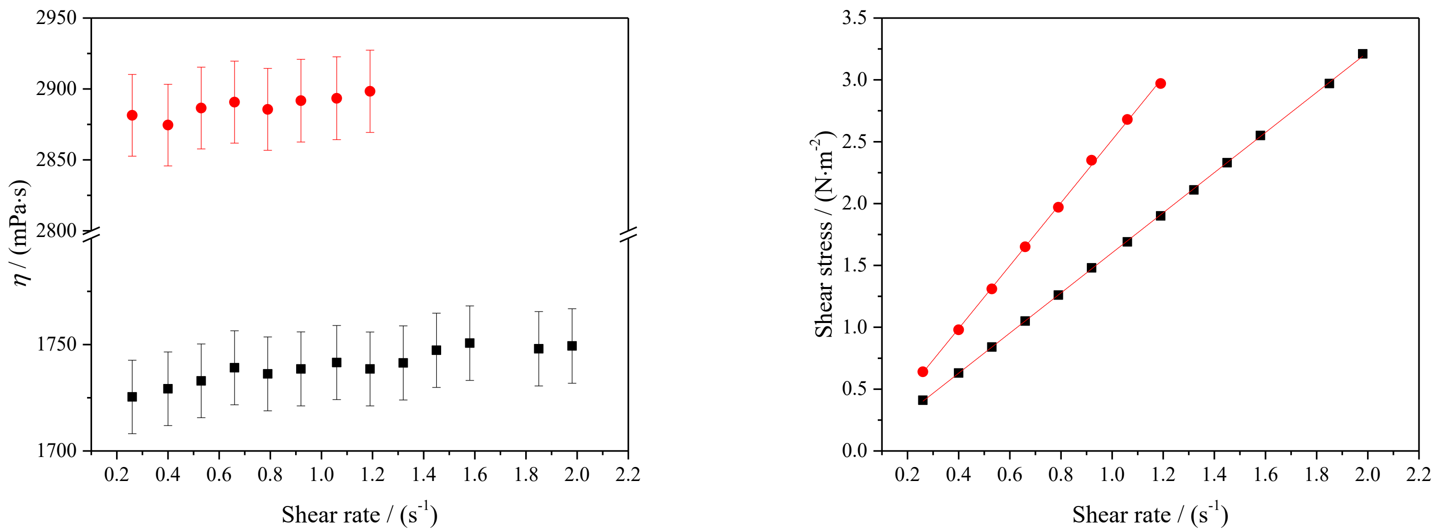

Figure S3. Verification of Newtonian behavior for pure DES and caffeine solution at m = 0.597 mol·kg^{-1} at T = 298.15 K: a) dynamic viscosity as a function of shear rate and b) shear stress as a function of shear rate.

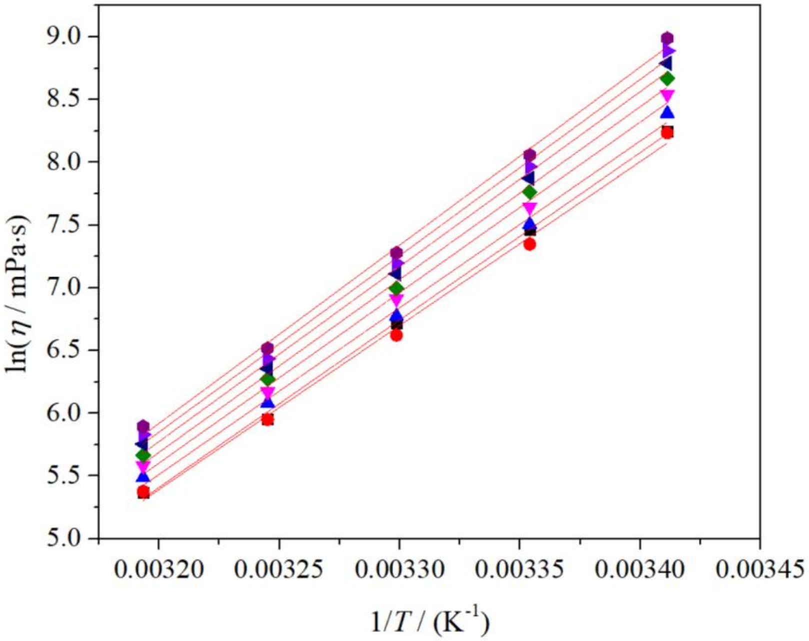

Figure S4. Dependence of lnη on reciprocal absolute temperature $(1/T)$ for the neat menthol–resorcinol DES and caffeine-containing systems at different caffeine molalities (m): = (■) 0.0000, (●) 0.100, (▲) 0.199, (▼) 0.300, (◆) 0.401, (◀) 0.501, (▶) 0.597 and (⬢) 0.701. The solid lines represent fits according to the Arrhenius equation (Eq. 16).

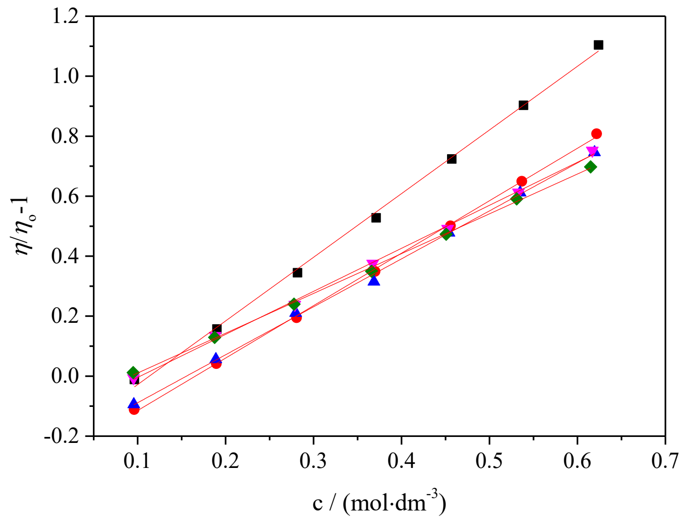

Figure S5. Plot of specific viscosity, $(\frac{\eta}{\eta_o}-1)$, against concentration, (c), of the caffeine + DES mixture, at different temperatures: T = (■) 293.15; (●) 298.15; (▲) 303.15; (▼) 308.15 and (◆) 313.15. The points represent experimental values and lines represent values calculated from Eq. (18).

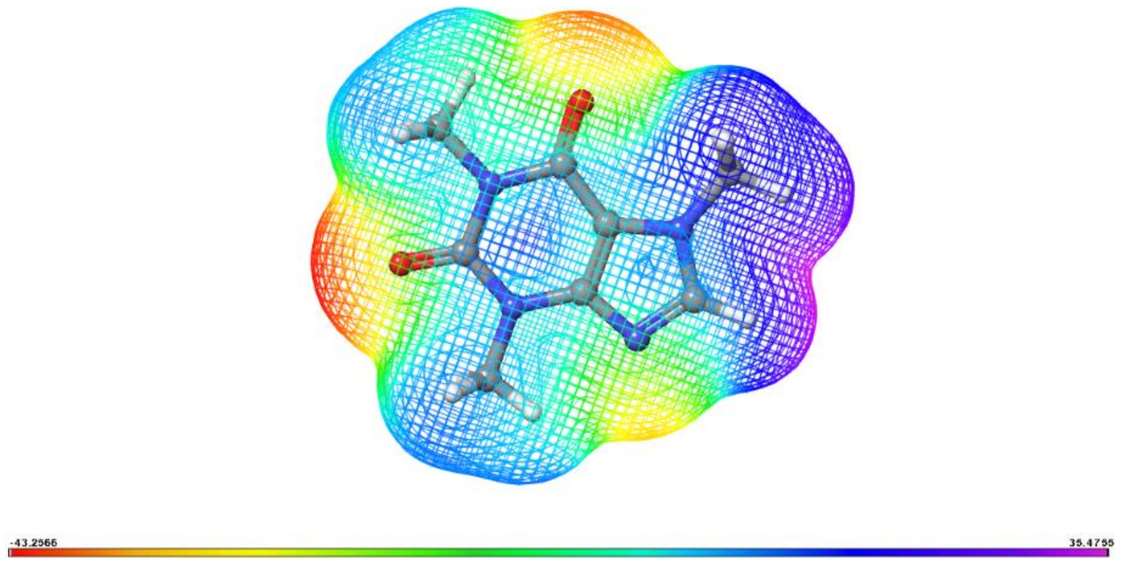

Figure S6. Molecular electrostatic potential surface of caffeine calculated at the B3LYP/6-311G(d,p) level of theory. Red and orange regions represent negative electrostatic potential, green regions indicate values close to zero, and blue and purple regions represent positive electrostatic potential.

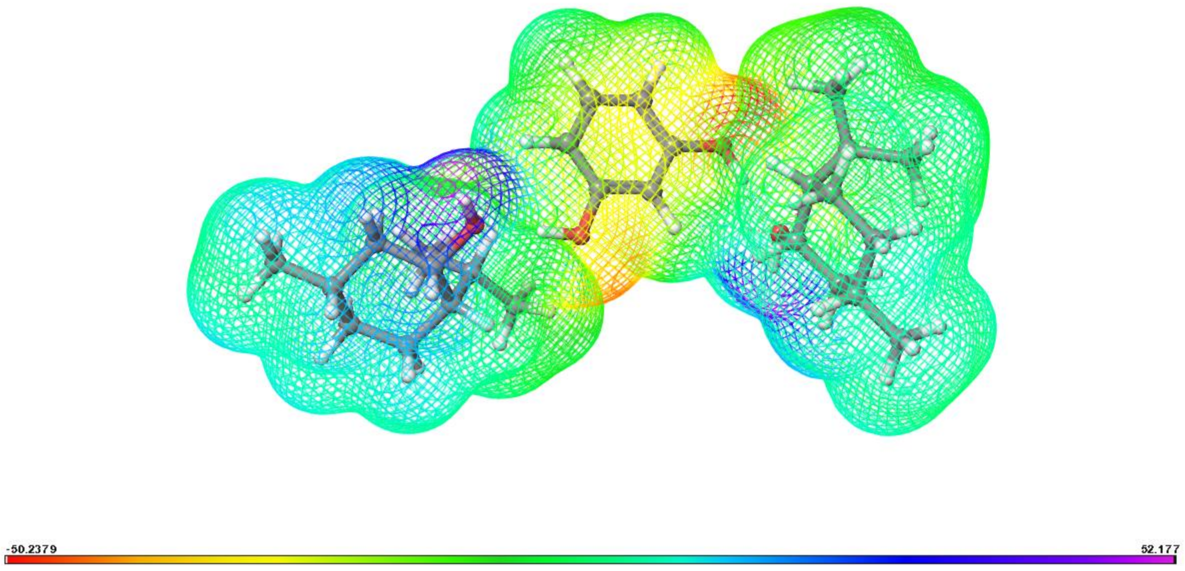

Figure S7. Molecular electrostatic potential surface of the representative DES cluster composed of two menthol molecules and one resorcinol molecule, calculated at the B3LYP/6-311G(d,p) level of theory. Red and orange regions represent negative electrostatic potential, green regions indicate values close to zero, and blue and purple regions represent positive electrostatic potential.

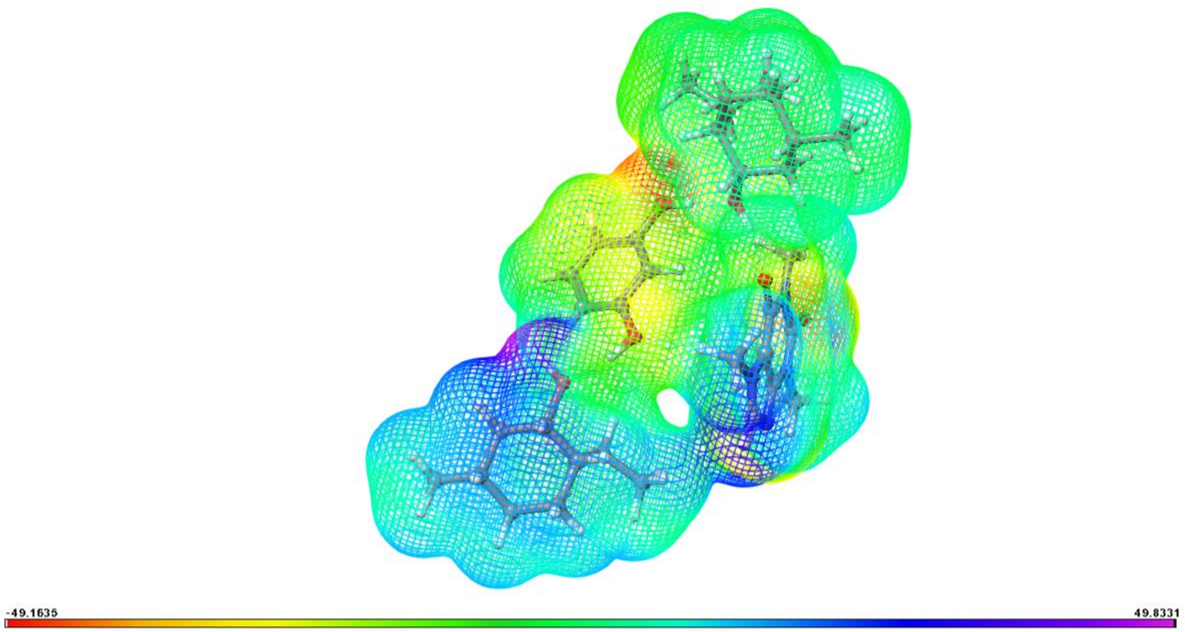

Figure S8. Molecular electrostatic potential surface of the representative cluster composed of two menthol molecules, one resorcinol molecule, and one caffeine molecule, calculated at the B3LYP/6-311G(d,p) level of theory. Red and orange regions represent negative electrostatic potential, green regions indicate values close to zero, and blue and purple regions represent positive electrostatic potential.
